# Restoring the Redox and Norepinephrine Homeostasis in Mouse Brains Promotes an Antidepressant Response

**DOI:** 10.1021/jacs.4c18046

**Published:** 2025-03-04

**Authors:** Qi Ding, Deqiang Li, Xin Zhang, Xue Xue, Ran Zhang, Di Su, Tony D. James, Ping Li, Xin Wang, Bo Tang

**Affiliations:** † College of Chemistry, Chemical Engineering and Materials Science, Key Laboratory of Molecular and Nano Probes, Ministry of Education, Collaborative Innovation Center of Functionalized Probes for Chemical Imaging in Universities of Shandong, Institutes of Biomedical Sciences, Shandong Normal University, Jinan 250014, P. R. China; ‡ Laoshan Laboratory, 168 Wenhai Middle Rd, Aoshanwei Jimo, Qingdao 266237, Shandong, P.R. China; § Department of Chemistry, University of Bath, Bath BA2 7AY, U.K.; ∥ School of Chemistry and Chemical Engineering, Henan Normal University, Xinxiang 453007, P. R. China; ⊥ College of Chemistry and Chemical Engineering, Northwest Normal University, Lanzhou 730070, P.R. China

## Abstract

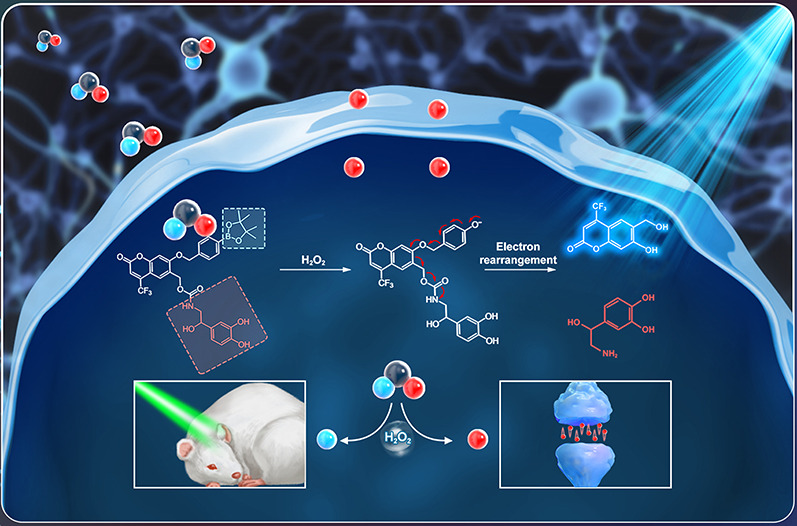

Effective diagnosis and treatment of major depressive disorder remains a major challenge because diagnostic criteria overlap with other conditions and 50% of patients are resistant to conventional treatments. Emerging evidence has indicated that oxidative stress and reduced norepinephrine are key pathological features of depression. Herein, we constructed a smart organic small-molecule fluorescence-based therapeutic system (Cou-NE-H_2_O_2_) for the diagnosis and treatment of depression targeted at restoring redox homeostasis and efficiently upregulating norepinephrine in the brain. Utilizing Cou-NE-H_2_O_2_, we could evaluate the depressive phenotype via the fluorescence monitoring of the redox state in mouse brains. By reducing hydrogen peroxide and continuously increasing norepinephrine, Cou-NE-H_2_O_2_ elicited a synergistic antidepressant action. Furthermore, we identified that Cou-NE-H_2_O_2_ can promote the expression of genes such as Grin2a, Drd1, and Fxyd2 related to the cyclic adenosine monophosphate signaling pathway, upregulate glutathione and cysteine to alleviate oxidative stress, and boost neuronal activity by enhancing dopaminergic synapses, ultimately achieving an effective antidepressant response. Taken together, this work provides a new strategy for the evaluation of depression and appropriate treatments and identifies the mechanisms underlying antioxidant and norepinephrine disorders in the brain as potential targets for the development of novel diagnostics and treatments for depression.

## Introduction

Depression, which has a high mortality rate, is a serious mental illness that poses a significant threat to human health.^1,2^ The low cure and high recurrence rate of depression causes great suffering to both the physical and mental health of patients.^3^ Currently, there is a lack of timely and accurate diagnostic standards as well as efficient treatment methods with minimal side effects suitable for depression. The diagnosis of depression in clinical practice mainly relies on a series of self-evaluation scales, which have strong subjectivity and can easily lead to inaccurate judgment of a patient’s condition by doctors, thereby affecting the delivery of appropriate treatments.^4,5^ At present, the treatment methods for depression include antidepressants, psychotherapy, modified electroconvulsive therapy, repeated transcranial magnetic stimulation, etc., in which psychotherapy, electrical or magnetic stimulation, and other methods have little therapeutic effect or the treatment process is exceptionally painful.^6^ Antidepressant drugs are widely used for clinical treatment, but there are some problems such as the long treatment period, poor effect, and lack of universality.^7^ Therefore, there is an urgent need to develop new strategies for timely, accurate diagnosis and effective drug treatment of depression.

Oxidative stress plays an undeniable role in the occurrence and development of depression.^8,9^ Reactive oxygen species (ROS), as the most direct biomarker for measuring oxidative stress levels, have been shown to play an extremely important role in the pathogenesis of depression.^10−12^ At the same time, the occurrence of depression is related to the low function of brain neurotransmitters.^13−15^ As an excitatory neurotransmitter in the brain, a decrease in the physiological level of norepinephrine (NE) can lead to low mood, listlessness, and other symptoms.^16,17^ Therefore, regulating the brain’s redox homeostasis and upregulating NE may provide new approaches for the development of effective strategies for the diagnosis and treatment of depression.

Fluorescence imaging technologies have become a powerful tool for clinical diagnosis and treatment due to their high sensitivity, high spatiotemporal resolution, real-time dynamics, and noninvasive nature.^18−23^ Integrated diagnostic and treatment reagents have great potential in visualizing disease markers, fluorescence-guided therapy, prognostic evaluation, etc., and have been applied to the treatment of tumors and neurological diseases.^24−26^ For example, a nanocomposite probe using photothermal and acoustic dynamic synergistic therapy was designed for the diagnosis and treatment of tumors,^24^ while an “activated” near-infrared II fluorescence nanodiagnostic and therapeutic system was constructed for the precise diagnosis and treatment of peritoneal metastases,^25^ and a new H_2_O_2_ responsive diagnostic and therapeutic nanoplatform was developed for the quantitative photoacoustic diagnosis and treatment of in vivo inflammation.^26^ The group of Wang has developed a light-responsive system that can effectively prevent the occurrence and development of inflammation-related depression.^27^ While the group of Zhang and Wang used black phosphorus nanosheets loaded with the antidepressant drug fluoxetine to shorten treatment times.^28^ In addition, they also developed a neural stem cell therapeutic strategy employing a nanoformulation to eliminate Aβ in mouse models and promote nerve regeneration.^29^ In recent years, small molecule fluorescent materials have become a hot area for the evaluation of the molecular mechanisms of brain diseases and the development of diagnostic and therapeutic reagents due to the advantages of stable composition, rapid metabolism, easy penetration through the blood-brain barrier, and low biological toxicity.^30−33^ However, small molecule fluorescent diagnostic and therapeutic reagents for depression are still extremely rare.

**Scheme 1:**
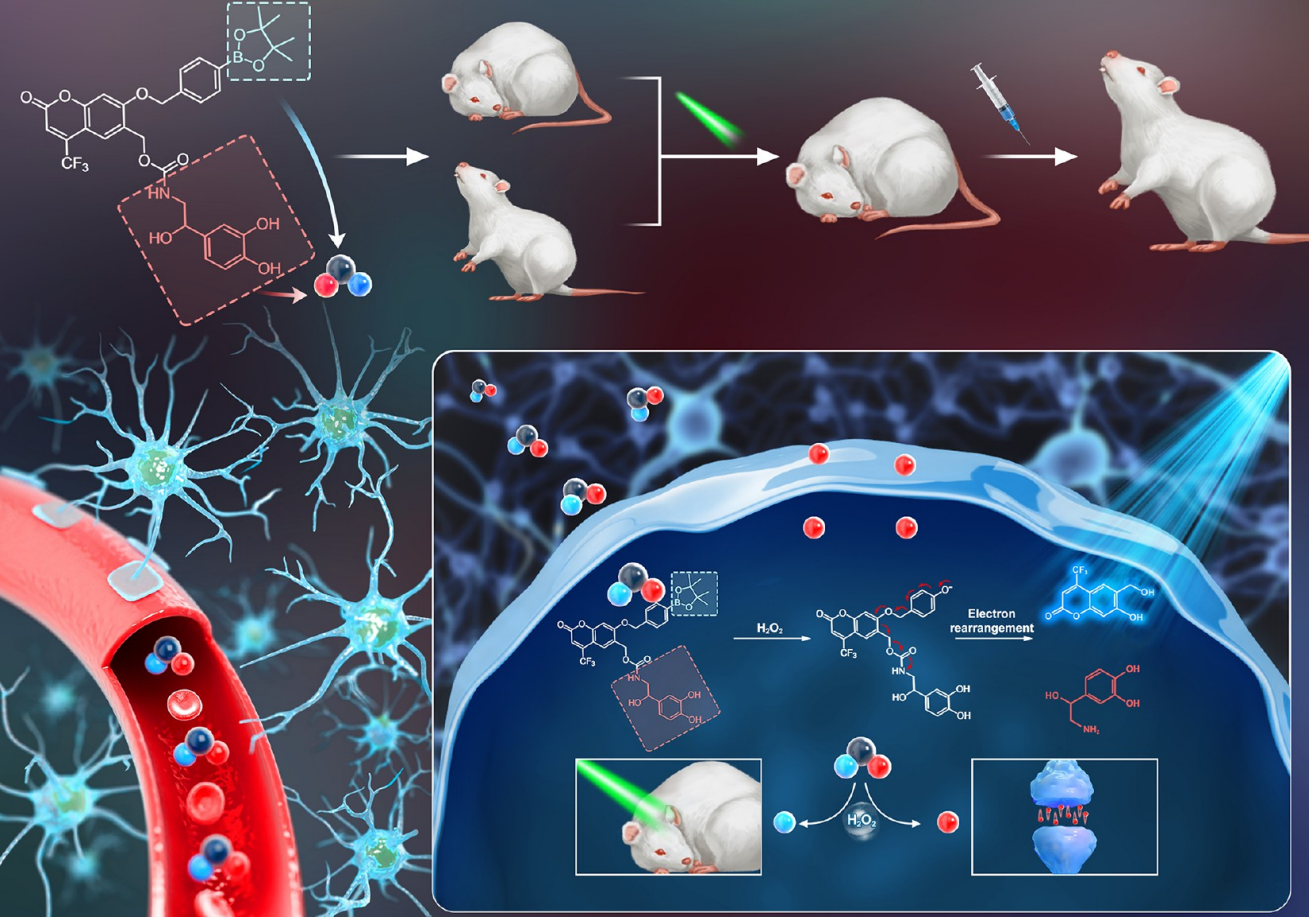
Schematic Diagram of the Antidepressant Diagnostic and Treatment Strategy Using Cou-NE-H_2_O_2_ for Alleviating Oxidative Stress and Upregulating Norepinephrine

**Figure 1:**
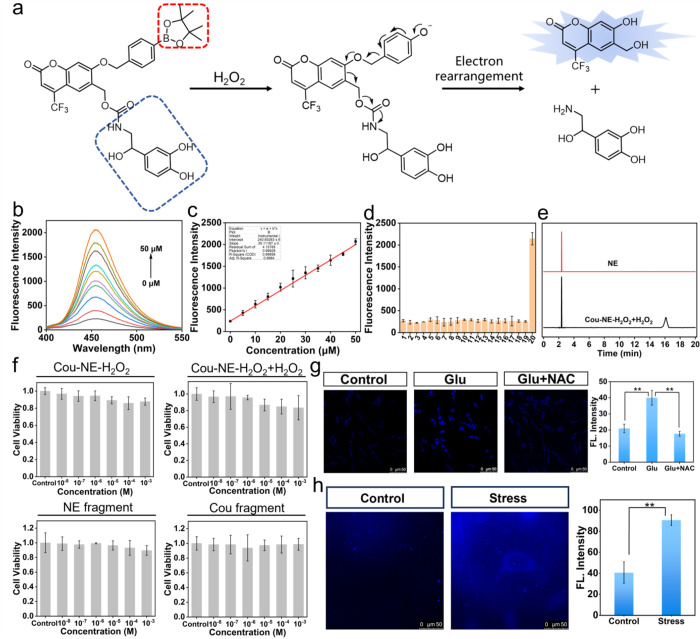
Recognition mechanism and photophysical properties of Cou-NE-H_2_O_2_. (a) Recognition mechanism of Cou-NE-H_2_O_2_ reacting with H_2_O_2_ and releasing NE. (b) One-photon fluorescence spectra of 20 μM Cou-NE-H_2_O_2_ after the addition of H_2_O_2_ (0–50 μM). (c) Linear correlation between the fluorescence intensities and H_2_O_2_ concentrations. (d) Fluorescence response of 20 μM Cou-NE-H_2_O_2_ to various ROS, RNS and metals (blank, 10 mM Na^+^, 10 mM K^+^, 100 μM Ca^2+^, 100 μM Ni^2+^, 100 μM Zn^2+^, 100 μM Fe^3+^,100 μM Fe^2+^, 100 μM Al^3+^, 100 μM Mg^2+^, 100 μM Mn^2+^, 100 μM Cu^2+^, 100 μM O_2_^•–^, 100 μM NO, 100 μM ^1^O_2_, 10 μM ONOO^–^, 100 μM •OH, 100 μM NaClO, 100 μM TBHP, 50 μM H_2_O_2_). (e) HPLC analysis of 20 μM Cou-NE-H_2_O_2_ reacting with 50 μM H_2_O_2_. Incubate at 37 °C for 20 min. (f) Cytotoxicity test of Cou-NE-H_2_O_2_ and its products after reaction with H_2_O_2_. (g) Fluorescence imaging of endogenous H_2_O_2_ in PC12 cells. Control: PC12 cells were incubated with 20 μM Cou-NE-H_2_O_2_ for 30 min. Glu: PC12 cells were preincubated with 10 mM glutamate for 12 h, and the cells were treated with 20 μM Cou-NE-H_2_O_2_ for 30 min. Glu + NAC: PC12 cells were preincubated with 10 mM glutamate for 12 h and loaded with 100 μM N-acetylcysteine (NAC) for 30 min, and the cells were treated with 20 μM Cou-NE-H_2_O_2_ for 30 min. Images were acquired using 405 nm for excitation, fluorescence emission windows is 440–480 nm. Scale bar = 50 μm. (h) fluorescence imaging in the brains of mice. Control: mice without drug stimulation. Stress: mice exposed to consecutive drug stimulation for 21 days. Images were acquired using 405 nm for excitation, fluorescence emission window is 440–480 nm. Scale bar = 50 μm. The data are expressed as mean ± SD, *n* = 3. **P < 0.01 compared to the control group.

To address the above issues, we propose a new strategy for the diagnosis and treatment of depression based on regulating brain redox homeostasis and efficiently upregulating NE (Scheme 1). Oxidative stress in the brain is a key pathological feature of depression. Hydrogen peroxide (H_2_O_2_) is the most abundant ROS, where fluctuations can directly represent the level of oxidative stress. Therefore, high concentrations of H_2_O_2_ in the brain of depression could be used to indicate the degree of oxidative stress, thus achieving the diagnosis of depression. Meanwhile, H_2_O_2_ could also serve as a target for depression treatment or a trigger for drug release. Coumarin with a high fluorescence quantum yield and anti-inflammatory properties was chosen as the multifunctional fluorophore. Based on this, a small molecule fluorescence diagnostic and therapeutic reagent Cou-NE-H_2_O_2_ was designed and developed for the accurate diagnosis and efficient treatment of depression. Cou-NE-H_2_O_2_ consists of three components: a fluorophore coumarin derivative, a phenylboronate ester unit with the ability to specifically recognize H_2_O_2_ and alleviate oxidative stress,^34^ and excitatory neurotransmitter NE. The phenylboronate ester unit and NE are connected to the coumarin derivative through monoether and carbonate bonds, respectively. Due to the intramolecular charge transfer (ICT) effect between the phenylboronate ester group and coumarin derivative, the fluorescence of coumarin is quenched. After reacting with H_2_O_2_, the phenylboronate ester group will be converted to a phenolate, followed by electron rearrangement, resulting in the cleavage of the carbonate and release of the NE and coumarin derivative, eliciting fluorescence recovery (Figure 1a).^35^ Fluorescence imaging experiments confirm that Cou-NE-H_2_O_2_ can reflect the levels of oxidative stress in the brain through changes in fluorescence intensity, thus achieving the early diagnosis of depression. Mouse behavioral and transcriptomic experiments indicated that, through the consumption of H_2_O_2_ and the release of NE, Cou-NE-H_2_O_2_ upregulates the signaling pathways such as cyclic adenosine monophosphate, dopaminergic synapse, glutathione, and cysteine metabolism, alleviating oxidative stress, improving mitochondrial function, and enhancing neuronal activity, resulting in the effective and efficient treatment of depression.

## Results and Discussion

### Synthesis and Characterization of Cou-NE-H_2_O_2_

The synthetic route for Cou-NE-H_2_O_2_ is shown in the Supporting Information. Optical properties of Cou-NE-H_2_O_2_ toward H_2_O_2_ under the simulated physiological conditions have been fully evaluated, including the absorption spectra, fluorescence emission spectra, linear fluorescence relationship, response specificity, pH environment, response kinetics and photostability (Figures 1b–d and S1–S7). The fluorescence of Cou-NE-H_2_O_2_ at 460 nm gradually enhanced as H_2_O_2_ was added in the range of 0 to 50 μM (Figure 1b). The linear relationship is *F* = 968.07 [H_2_O_2_ (μM) + 159.15], with a correlation coefficient of 0.997 (Figure 1c). The NE release efficiency of Cou-NE-H_2_O_2_ was evaluated to be 69% in vitro through High-Performance Liquid Chromatography (HPLC) (Figure 1e). In addition, the biotoxicity of Cou-NE-H_2_O_2_ and the products after reaction with H_2_O_2_ were determined, confirming that Cou-NE-H_2_O_2_ and its reaction products exhibit minimal biotoxicity (Figure 1f). The above results indicate that Cou-NE-H_2_O_2_ can be quantitatively activated by H_2_O_2_ and release NE in cells in vivo.

### Ability of Cou-NE-H_2_O_2_ To Monitor Oxidative Stress in Living Systems

Experiments to determine whether Cou-NE-H_2_O_2_ could be used for the fluorescence imaging of endogenous H_2_O_2_ were conducted by using fluorescence confocal microscopy. First, PC12 cells were incubated with a high concentration of glutamate (10 mM) for 12 h,^36,37^ and then, the cells were incubated with Cou-NE-H_2_O_2_. As shown in Figure 1g, the fluorescence intensity of Cou-NE-H_2_O_2_ in the cells pretreated with glutamate was significantly higher than that in the control group, indicating that high levels of H_2_O_2_ were produced in the cells after glutamate treatment. To demonstrate that the change in fluorescence intensity for Cou-NE-H_2_O_2_ was indeed caused by the change in the H_2_O_2_ level, N-acetylcysteine (NAC, a H_2_O_2_ scavenger) was added to PC12 cells after pretreatment with glutamate.^38^ After the addition of NAC, the fluorescence intensity of Cou-NE-H_2_O_2_ in cells significantly decreased, confirming that the change in fluorescence intensity of Cou-NE-H_2_O_2_ was caused by changes in the H_2_O_2_ concentration (Figure 1g). The above experimental results indicate that Cou-NE-H_2_O_2_ can specifically recognize H_2_O_2_ and monitor the fluctuations of H_2_O_2_ levels in live cells in situ and in real time.

Then, the ability of Cou-NE-H_2_O_2_ to monitor changes in H_2_O_2_ concentrations in the brains of normal and depressed mice was evaluated. After being administered with corticosterone (CORT),^39^ behavioral tests including a sucrose preference test, forced swimming experiment, tail suspension experiment, and open field experiment were used to confirm that a depression model for mice was successfully established. Next, the mice were intraperitoneally injected with 0.34 mg kg^–1^ Cou-NE-H_2_O_2_, and 30 min later, the mice were sacrificed, followed by a fluorescence imaging experiment on mouse brain tissue slices. Fluorescence imaging results indicated that the fluorescence intensity for the brains of depressive phenotype mice was significantly higher than that of normal mice, confirming a significant increase in H_2_O_2_ levels in the brains of depressed mice (Figure 1h). These results illustrate that Cou-NE-H_2_O_2_ can specifically recognize the overexpressed H_2_O_2_ in the mouse brain, visualize the degree of oxidative stress, and accurately diagnose depression according to the different contents of H_2_O_2_ in the brains.

### Optimization of Treatment Strategies of Cou-NE-H_2_O_2_

**Figure 2:**
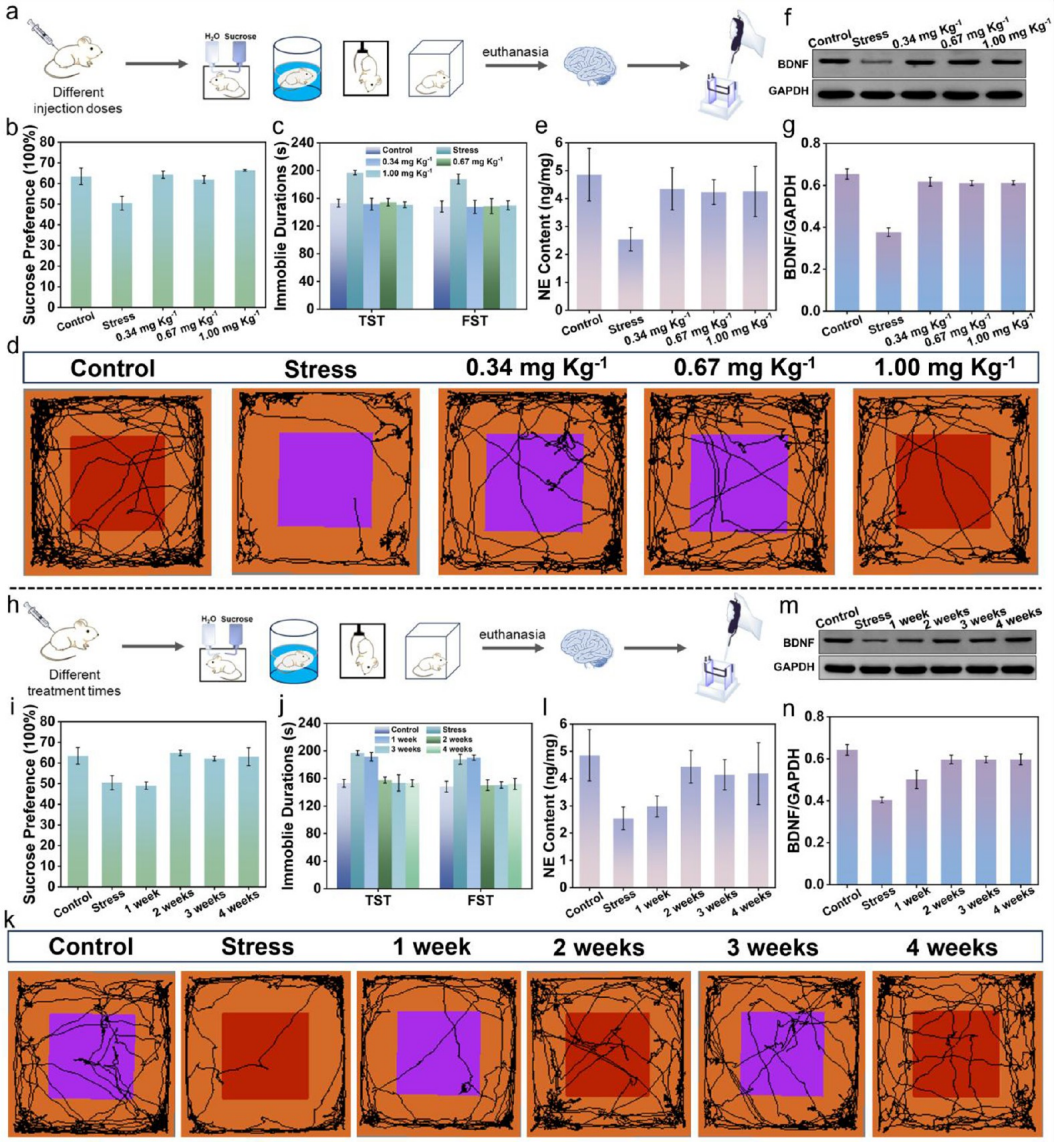
Optimal strategies for injection doses and treatment times of Cou-NE-H_2_O_2_. (a) Experimental diagram of depressed mice after injection of different doses of Cou-NE-H_2_O_2_. (b) Sucrose preference test of depressed mice injected with 0.34, 0.67, and 1.00 mg kg^–1^ doses of Cou-NE-H_2_O_2_, respectively. (c) Immobile durations of depressed mice injected with 0.34, 0.67, and 1.00 mg kg^–1^ doses of Cou-NE-H_2_O_2_ in tail suspension test and forced swimming test, respectively. (d) Open field tracks of mice injected with 0.34, 0.67, and 1.00 mg kg^–1^ doses of Cou-NE-H_2_O_2_, respectively. (e) ELISA assay kit of NE in depressed mice injected with 0.34, 0.67, and 1.00 mg kg^–1^ doses of Cou-NE-H_2_O_2_. (f, g) BDNF contents of depressed mice injected with 0.34, 0.67, and 1.00 mg kg^–1^ doses of Cou-NE-H_2_O_2_, respectively. (h) Experimental diagram of depressed mice injected with 0.34 mg kg^–1^ Cou-NE-H_2_O_2_ for different treatment times. (i) Sucrose preference test of depressed mice injected with 0.34 mg kg^–1^ Cou-NE-H_2_O_2_ for 1 week, 2 weeks, 3 weeks and 4 weeks, respectively. (j) Immobile durations of depressed mice injected with 0.34 mg kg^–1^ Cou-NE-H_2_O_2_ for 1 week, 2 weeks, 3 weeks and 4 weeks in tail suspension test and forced swimming test, respectively. (k) Open field tracks of mice injected with 0.34 mg kg^–1^ Cou-NE-H_2_O_2_ for 1 week, 2 weeks, 3 weeks and 4 weeks, respectively. (l) ELISA assay kit of NE in depressed mice injected with 0.34 mg kg^–1^ Cou-NE-H_2_O_2_ for 1 week, 2 weeks, 3 weeks and 4 weeks. (m, n) BDNF contents of depressed mice injected with 0.34 mg kg^–1^ Cou-NE-H_2_O_2_ for 1 week, 2 weeks, 3 weeks, and 4 weeks, respectively. The data are expressed as mean ± SD, *n* = 3.

The fluorescence imaging results of cells and in vivo indicated that Cou-NE-H_2_O_2_ can respond specifically to H_2_O_2_ in living organisms and can distinguish normal mice from mice with depression phenotypes according to different levels of oxidative stress. Therefore, we further investigated whether Cou-NE-H_2_O_2_ has antidepressant effects. First, the dosage of Cou-NE-H_2_O_2_ used was optimized. Cou-NE-H_2_O_2_ in doses of 0.34, 0.67, and 1.00 mg kg^–1^ was intraperitoneally injected into depressed mice for 2 consecutive weeks to monitor the depression-like behavior of the mice (Figure 2a). As shown in Figure 2b–d, depressed mice injected with 0.34 mg kg^–1^ Cou-NE-H_2_O_2_ exhibited relief of depression-like behavior similar to the levels observed for normal mice after 2 weeks of treatment, indicating that 0.34 mg kg^–1^ Cou-NE-H_2_O_2_ has a significant antidepressant effect.

To verify that Cou-NE-H_2_O_2_ can release NE after reacting with H_2_O_2_, we monitored the content of NE in the brains of mice using an ELISA Kit. As shown in Figure 2e, it was found that after 2 weeks of treatment, the NE content in the brain of depressed mice injected with 0.34 mg kg^–1^ Cou-NE-H_2_O_2_ significantly increased compared to depressed mice and was close to that for normal mice. The above experimental results indicate that a dose of 0.34 mg kg^–1^ of Cou-NE-H_2_O_2_ exhibits good antidepressant effects.

Brain-derived neurotrophic factor (BDNF) is a protein that plays a crucial function in the growth, development, and maintenance of brain neurons.^40^ Studies have shown that its expression level is closely related to various mental disorders such as depression.^33,41,42^ In order to further confirm the antidepressant effect of Cou-NE-H_2_O_2_, we measured the BDNF content in the brains of mice using a Western Blot experiment. It was found that after 2 weeks of treatment, the content of BDNF in the brain of depressed mice injected with 0.34 mg kg^–1^ Cou-NE-H_2_O_2_ was similar to the standard for normal mice (Figure 2f,g). Considering the biological toxicity results for Cou-NE-H_2_O_2_ and other factors, we chose 0.34 mg kg^–1^ of Cou-NE-H_2_O_2_ for the subsequent experiments.

After the dosage of Cou-NE-H_2_O_2_, the treatment time of mice was also optimized. Depressed mice were treated with 0.34 mg kg^–1^ Cou-NE-H_2_O_2_ for 1 week, 2 weeks, 3 weeks, and 4 weeks, respectively. Subsequently, the depression-like behavior of the mice, BDNF content, and NE content in mice brains were evaluated (Figure 2h). As shown in Figure 2i–k, there was no significant improvement in depression-like behavior for mice when the treatment lasted for 1 week. However, when the treatment time was increased to 2 weeks, the depression-like behavior of the mice was distinctly improved and remained constant with an extension of treatment time. Then, the NE and BDNF levels in the brains of the mice were also measured separately. The results indicated that consistent with the trend of depression-like behavior changes in mice, there was only a slight increase in NE and BDNF levels at 1 week of treatment (Figure 2l–n). However, when the treatment time increased to 2 weeks, the content of NE and BDNF significantly enhanced and approached the levels found for normal mice, and as the treatment time increased, the content of NE and BDNF was constant. The above experimental results indicate that when using Cou-NE-H_2_O_2_ to treat depressed mice for 2 weeks, Cou-NE-H_2_O_2_ exhibits obvious antidepressant effects. Therefore, in the subsequent experiments, we chose a treatment time of 2 weeks. The above experimental results further confirmed that Cou-NE-H_2_O_2_ exhibits an excellent antidepressant effect with fast onset and at a low dose.

### Cou-NE-H_2_O_2_ Successfully Penetrates the BBB To Exert a Highly Efficient Antidepressant Effect

**Figure 3:**
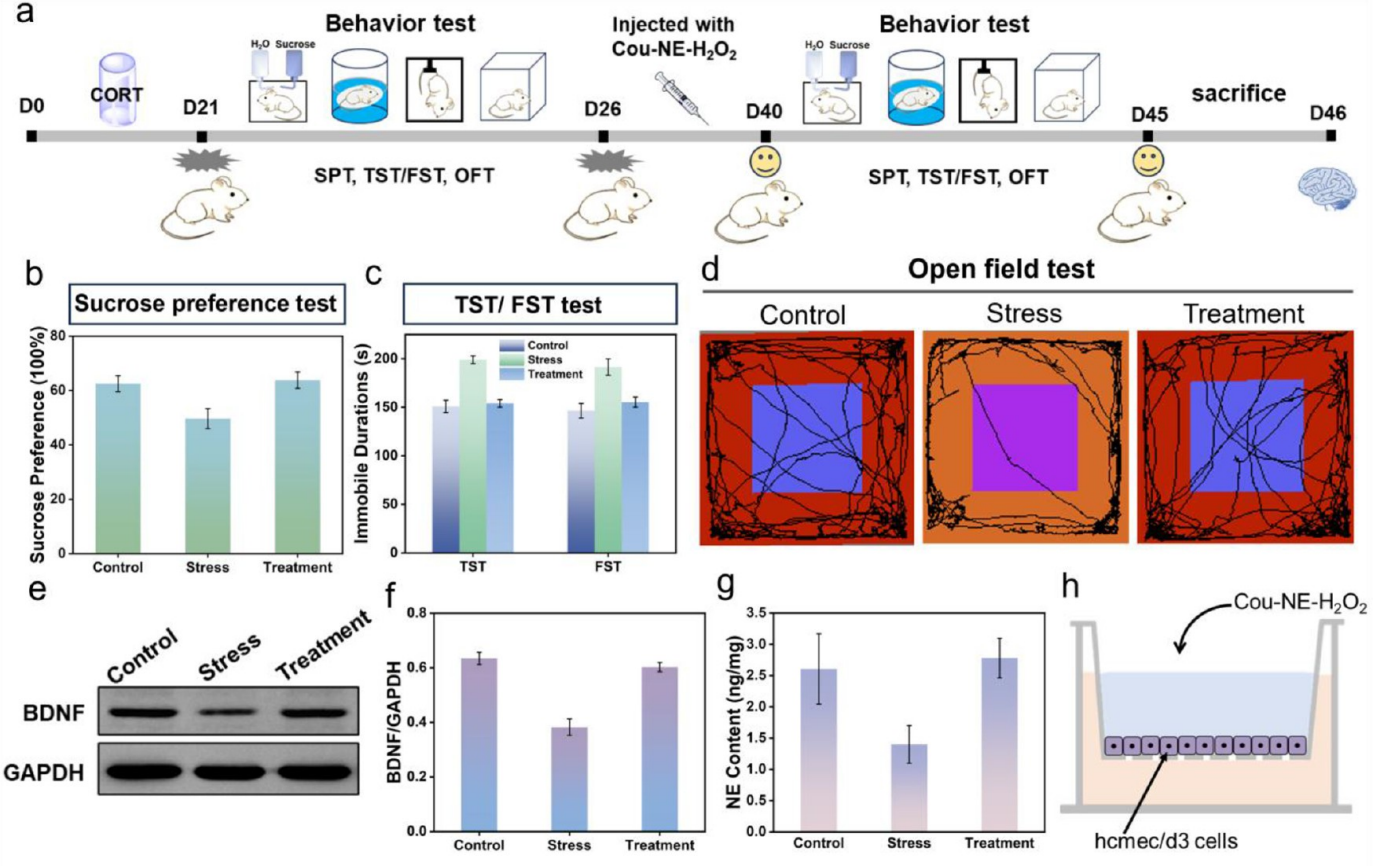
Cou-NE-H_2_O_2_ successfully penetrates the BBB to exert a highly efficient antidepressant effect. (a) Representative experimental procedures of the depressed mice model established by CORT and treatment by Cou-NE-H_2_O_2_ in depressed mice. (b) Sucrose preference test of mice. Control: control mice. Stress: mice treated with CORT. Treatment: depressed mice injected with Cou-NE-H_2_O_2_. (c) Immobile durations of mice in the tail suspension test and forced swimming test. (d) Open field tracks of mice. (e, f) BDNF contents of mice brains. (g) ELISA assay kit of NE in mice. (h) Schematic illustration of the in vitro BBB model. The data are expressed as mean ± SD, *n* = 3.

After a series of preliminary experiments to evaluate and optimize the therapeutic dose and treatment duration of Cou-NE-H_2_O_2_, the high efficiency and low toxicity of Cou-NE-H_2_O_2_ confirm that it could be used as an antidepressant (Figure S11). Therefore, we evaluated the antidepressant effect of Cou-NE-H_2_O_2_ with a treatment dose of 0.34 mg kg^–1^ and a treatment time of 2 weeks. First, mice with a depression phenotype were modeled using CORT, and then the depressed mice were divided into two groups. One group was treated with Cou-NE-H_2_O_2_, as the treatment group, and the other group was fed CORT to maintain the mouse depression phenotype, as the depression group. After intraperitoneal injection of 0.34 mg kg^–1^ Cou-NE-H_2_O_2_ and continuous treatment for 2 weeks, the therapeutic effect was assessed by estimating the depression-like behavior, NE, and BDNF levels in the brains of the mice (Figure 3a). As shown in Figure 3b–d, compared with the depression group, the proportion of sucrose intake of mice in the treatment group was higher, and the immobile time in the tail suspension and forced swimming experiments was reduced; the frequency of mice going to the center of the open field experiment was increased, and their behavior level was basically the same as that of normal mice, indicating that Cou-NE-H_2_O_2_ had obvious antidepressant effects. At the same time, the content of BDNF in the brains of the control group mice, depression group mice, and treatment group mice was measured using Western Blot experiments. It was found that the BDNF content in the brains of the depression group mice was the lowest (Figure 3e,f). After 2 weeks of treatment, the BDNF content in the brains of the treatment group mice significantly increased and its expression level was consistent with that of the control group mice. The results indicate that Cou-NE-H_2_O_2_ can promote the expression of BDNF in mice brains and heighten the synergistic effect of the antidepressant. Similarly, an ELISA Kit was used to evaluate the NE content in the brain of mice, and it was found that the NE content in the depression group was the lowest; after treatment, the NE content of the mice brains was increased significantly and was close to the level of mice in the control group (Figure 3g). These results illustrate that Cou-NE-H_2_O_2_ can effectively upregulate the NE content in the brain of mice with a depression phenotype to maintain the activity of excitatory neurons and enhance the antidepressant effect. In addition, we simulated the blood-brain barrier in vitro and evaluated the ability of Cou-NE-H_2_O_2_ to penetrate the BBB,^27^ illustrating that Cou-NE-H_2_O_2_ can indeed penetrate the BBB and exert antidepressant effects in brain regions (Figures 3h and S9). The above experimental results indicate that a 0.34 mg kg^–1^ treatment regimen for 2 weeks by Cou-NE-H_2_O_2_ in depressed mice exhibits excellent antidepressant effects.

### Biotoxicity and Transcriptomic Analysis of Cou-NE-H_2_O_2_ in Mice

**Figure 4:**
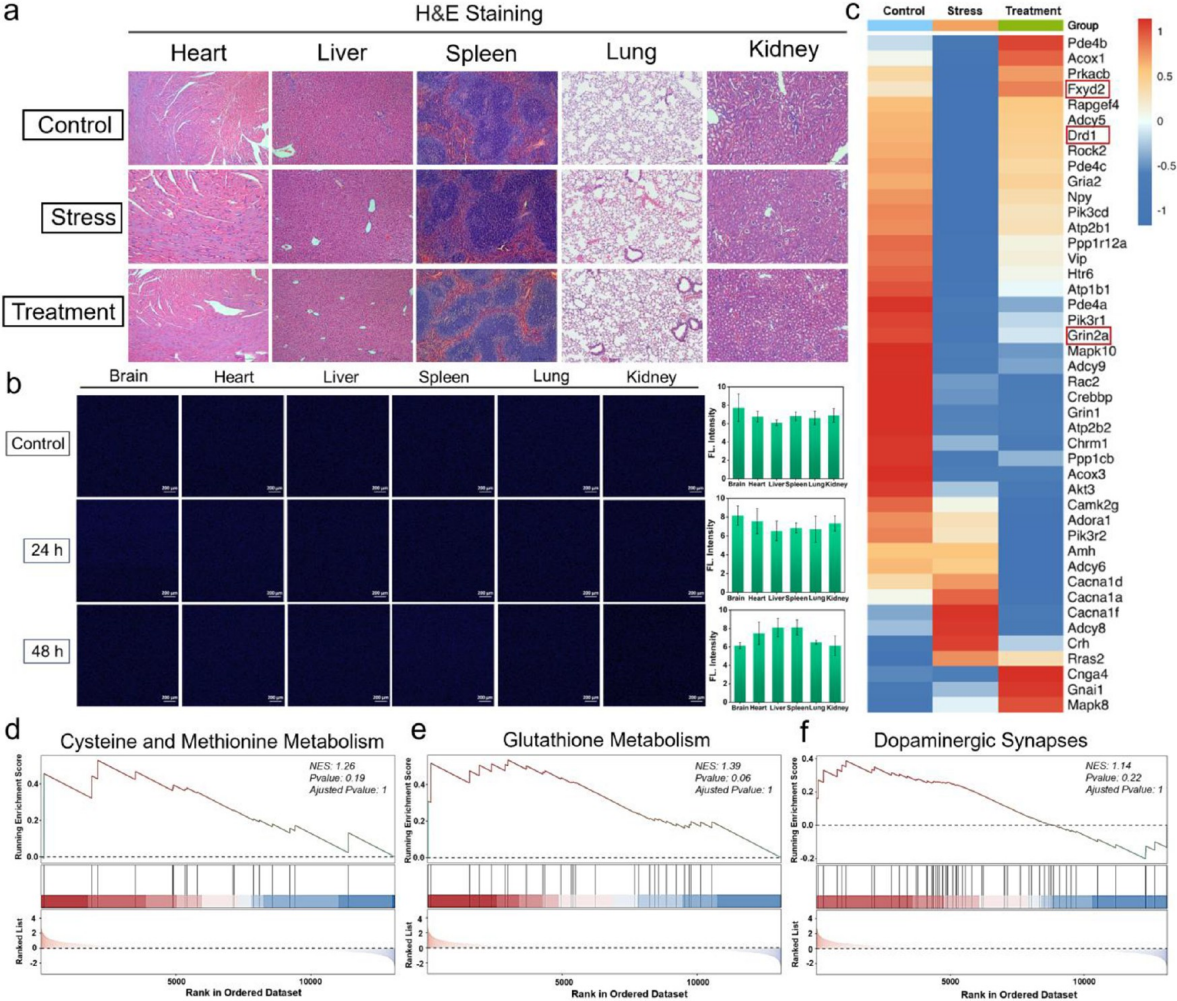
Biotoxicity and transcriptomic analysis of Cou-NE-H_2_O_2_ in mice. (a) Hematoxylin–eosin staining (H&E staining) of the lung, liver, spleen, kidney and heart after mice continuously injected with Cou-NE-H_2_O_2_ for 2 weeks. (b) Fluorescence imaging of the brain, lung, liver, spleen, kidney and heart of mice after injection with Cou-NE-H_2_O_2_ 24/48 h. TP excitation is 800 nm, and the emission window is 440–480 nm. Scale bar: 200 μm. The data are expressed as mean ± SD, *n* = 3. (c) Heatmap of differential expressed genes in the cyclic adenosine monophosphate signaling pathway (red indicates relatively high expressed genes, blue indicates relatively low expressed genes). (d) Comparative analysis of gene sets of cysteine and methionine metabolism in Cou-NE-H_2_O_2_ treated mice and depressed mice. (e) Comparative analysis of gene sets of glutathione metabolism in Cou-NE-H_2_O_2_ treated mice and depressed mice. (f) Comparative analysis of gene sets of dopaminergic synapses in Cou-NE-H_2_O_2_ treated mice and depressed mice. Each experiment was repeated three times.

In order to further confirm the biological safety of Cou-NE-H_2_O_2_, H&E staining experiments were carried out. According to the staining of tissue sections of five major organs, namely, lung, liver, spleen, kidney, and heart, Cou-NE-H_2_O_2_ has very low biological toxicity and has almost no effect on the organs of mice (Figure 4a). Likewise, we also investigated the metabolism of Cou-NE-H_2_O_2_. After intraperitoneal injection of 0.34 mg kg^–1^ Cou-NE-H_2_O_2_ for 24 h/48 h, fluorescence imaging was performed on major organs such as the brain, lung, liver, spleen, kidney, and heart. It was found that almost all of these organs displayed no fluorescence, indicating that Cou-NE-H_2_O_2_ did not remain and aggregate within these organs (Figure 4b). The experimental results indicated that Cou-NE-H_2_O_2_ could be rapidly metabolized.

Next, we explored the pathway through which Cou-NE-H_2_O_2_ exerts its antidepressant effect and determined the genetic differences in depressed mice before and after treatment through transcriptomic analysis. The results revealed that Cou-NE-H_2_O_2_ significantly upregulated the expression levels of Grin2a, Drd1, and FXYd2 genes in the cyclic adenosine monophosphate signaling pathway, thereby maintaining the normal physiological function and mitochondrial function of cells (Figures 4c and S13). Meanwhile, we also discovered that Cou-NE-H_2_O_2_ can upregulate the cysteine and methionine metabolism, glutathione metabolism, and dopaminergic synapse pathways in the brains of depressed mice (Figure 4d–f). The experimental results also confirmed that Cou-NE-H_2_O_2_ achieves an antidepressant effect by alleviating oxidative stress, enhancing mitochondrial function, and maintaining neuronal activity. These experimental results further improve the pathogenesis of depression and offer new targets for the diagnosis and treatment of depression, proving that depression is closely related to mitochondrial function and oxidative stress, and demonstrating the effectiveness of our designed diagnosis and treatment strategy at the molecular mechanistic level.

Depression is a kind of mental illness with a particularly high disability and death rate, which is due to the lack of accurate diagnosis and effective treatments.^1,3^ Oxidative stress and low levels of excitatory neurotransmitters in the brain are considered pivotal factors in the pathogenesis of depression.^8,9,13,15^ Therefore, with the goal of regulating the redox state and upregulating excitatory neurotransmitters in the brain, we designed and developed fluorescent diagnostic and therapeutic Cou-NE-H_2_O_2_ for the diagnosis and treatment of depression. Cou-NE-H_2_O_2_ is composed of a coumarin derivative, a phenylboronate ester that can specifically recognize H_2_O_2_^34^ and norepinephrine. After penetrating the blood-brain barrier, Cou-NE-H_2_O_2_ reacts with H_2_O_2_ to release the coumarin derivative and NE in an oxidative environment within the cells. By combining fluorescence imaging technology and behavioral evaluation, we were able to precisely diagnose depression using changes in the fluorescence intensity of Cou-NE-H_2_O_2_, as well as provide efficient treatment of depression by upregulating NE in the mouse brains and alleviating oxidative stress. Significantly, transcriptomic experiments used to explore the antidepressant targets of Cou-NE-H_2_O_2_ enhanced our understanding of the molecular mechanisms of depression.

At present, the diagnosis of depression in clinical practice mainly relies on a series of self-assessment scales completed independently by patients, combined with the doctors’ comprehensive evaluation of the patients, to make the diagnosis of depression.^4,5^ This diagnostic method has strong subjectivity and relies on the doctor’s medical experience to a great extent, which can lead to inaccurate judgment of the patient’s condition by the doctor, thereby hindering subsequent antidepressant treatment. Therefore, the focus of our proposed diagnosis and treatment strategy is to assess the degree of depression using imaging based on changes in fluorescence intensity, thereby achieving an accurate diagnosis of depression. Considering the severe redox imbalance in the body of patients with depression, ROS can serve as a biomarker of oxidative stress, and the changes in levels may indicate the severity of depression. Our as-developed Cou-NE-H_2_O_2_ can penetrate the blood-brain barrier, visually monitor the levels of H_2_O_2_ in a mouse brain, and accurately diagnose depression by evaluating the different levels of H_2_O_2_ in the brains of normal and depressed mice using fluorescence imaging technology. Compared with the self-assessment scale, this diagnostic strategy can intuitively observe the severity of depression, avoid interference from subjective judgments, and establish a reliable and true diagnostic standard for depression. In addition, on the basis of different levels of H_2_O_2_, the fluorescence characteristics of Cou-NE-H_2_O_2_ could also be used to guide clinical medication and provide an appropriate prognosis for depression.

Another highlight of this work is the development of a treatment method for depression that exhibits good efficacy, fast onset, and minimal side effects. Until now, the clinical treatment of depression mainly includes antidepressant drugs, psychotherapy, modified electroconvulsive therapy, repeated transcranial magnetic stimulation, etc.^6^ Antidepressants are the conventional treatment, but there are some problems such as slow onset (usually one month), obvious side effects (nausea and vomiting), and poor universality;^7^ psychotherapy is primarily aimed at mild depression but has little effect on moderate or severe depression;^43^ and electrical or magnetic stimulation can cause memory loss, headaches, cognitive dysfunction, and other side effects.^44^ Hence, there is an urgent need to develop depression treatments with rapid effects exhibiting minimal side effects.

In view of the germane relation between the pathogenesis of depression and oxidative stress and low levels of excitatory neurotransmitters, a synergistic strategy for regulating redox states in the brain and upregulating excitatory neurotransmitter levels may become a new direction for the treatment of depression. In order to maximize the antidepressant effect of Cou-NE-H_2_O_2_ with minimal side effects, the dosage and treatment time of Cou-NE-H_2_O_2_ were optimized. Through behavioral tests such as sucrose preference test, forced swimming test, tail suspension test, and open field test, it was found that after 2 weeks of intraperitoneal injection with 0.34 mg kg^–1^ Cou-NE-H_2_O_2_, the sucrose intake and immobility time of depressed mice returned to normal levels, indicating that Cou-NE-H_2_O_2_ has a fast and efficient antidepressant effect. Beyond that, Western Blot experiments and ELISA Kit evaluation indicated a significant increase in BDNF and norepinephrine levels in the brains of depressed mice after treatment, illustrating that Cou-NE-H_2_O_2_ can promote BDNF expression and upregulate norepinephrine levels in the brain. These experimental results confirm that Cou-NE-H_2_O_2_ can realize the efficient treatment of depression by consuming H_2_O_2_ to mitigate oxidative stress and efficiently boost norepinephrine. Compared to traditional antidepressant drugs with a one-month treatment cycle, Cou-NE-H_2_O_2_ reduces the treatment time to 2 weeks, greatly reducing the patient’s pain and economic burden. In addition, when evaluating the side effects of Cou-NE-H_2_O_2_, we observed and recorded physiological indicators such as food intake, hair, weight, and blood pressure in mice. Notably, it was found that there were no significant side effects in mice treated with Cou-NE-H_2_O_2_, confirming that Cou-NE-H_2_O_2_ as an antidepressant reagent exhibits low biological toxicity and minimal side effects. In summary, the above experimental results confirm that Cou-NE-H_2_O_2_ is an effective treatment for depression with the advantages of rapid onset, good efficacy, and minimal side effects. Notably, as an organic small molecule fluorescent material, Cou-NE-H_2_O_2_ also possesses the merits of stable composition and rapid metabolism, easy penetration of the blood-brain barrier, and low biological toxicity. As such, we anticipate that Cou-NE-H_2_O_2_ will provide an integrated fluorescent diagnostic and therapeutic approach for the treatment of depression.

More importantly, this study investigated the antidepressant mechanism of Cou-NE-H_2_O_2_. Using gene transcriptomics experiments, it was found that Cou-NE-H_2_O_2_ promotes mitochondrial functions by upregulating the expression levels of Grin2a, Drd1, and FXYd2 genes in the cyclic adenosine monophosphate signaling pathway. Meanwhile, Cou-NE-H_2_O_2_ can comprehensively upregulate cysteine and methionine and glutathione metabolism signaling pathways to alleviate oxidative stress and maintain neural cell physiological function by enhancing the dopaminergic synapses signaling pathway. The experimental results indicate that the antidepressant effects of Cou-NE-H_2_O_2_ are caused by relieving oxidative stress and improving mitochondrial function. These experimental results uncover potential new therapeutic targets for depression for the first time, enabling a better understanding of the molecular mechanism of depression, and affording a factual basis for the development of fluorescent diagnostic and therapeutic reagents for depression.

## Conclusions

In order to fill the gap in the diagnosis and treatment of depression, based on the high levels of oxidative stress and low levels of excitatory neurotransmitters in the brain during the occurrence and development of depression, we propose a new strategy for depression diagnosis and treatment that regulates the redox state in the brain and efficiently upregulates norepinephrine. We have constructed the fluorescent diagnostic and therapeutic reagent Cou-NE-H_2_O_2_ for the precise diagnosis and treatment of depression. Cou-NE-H_2_O_2_ can specifically recognize overexpressed H_2_O_2_ in the brains of depressed mice, assess the degree of depression by fluorescence imaging, and achieve an accurate diagnosis of depression. On the basis of alleviating oxidative stress, the controlled release of norepinephrine can upregulate the level of excitatory neurotransmitters in the brain, maintain the normal physiological function of excitatory neurons, and attain efficient and collaborative treatment of depression. More importantly, transcriptomic experiments reveal the antidepressant mechanism of Cou-NE-H_2_O_2_ for the first time: achieving antidepressant effects by upregulating the cyclic adenosine monophosphate, tricarboxylic acid cycle, glutathione, and cysteine signaling pathways. Compared with traditional antidepressants, Cou-NE-H_2_O_2_ has the advantages of fast onset, good efficacy, and minimal side effects and as such is expected to provide a new generation of fluorescent diagnostic and therapeutic reagents suitable for the treatment of depression. This work proposes new strategies for the development of diagnostic and treatment regimens for depression, providing new approaches and potential targets for understanding the pathogenesis of depression.

## Supporting Information Available

Additional experimental data including experimental details; synthesis of Cou-NE-H_2_O_2_; photophysical properties; and fluorescence imaging (PDF)
