# Supplementary material for: Restoring the Redox and Norepinephrine Homeostasis in Mouse Brains Promotes an Antidepressant Response
Source: J Am Chem Soc. 2025 Mar 4;147(13):11239–49. doi: 10.1021/jacs.4c18046 (PMC11969533; doi:10.1021/jacs.4c18046)
Supplement: Supplementary file 1 — ja4c18046_si_001.pdf [file ja4c18046_si_001.pdf]

Electronic Supplementary Information (ESI) for Journal of the American Chemical Society.

Supporting Information

Restoring the Redox and Norepinephrine Homoeostasis in Mouse Brains Promotes an Antidepressant Response

Qi Ding,[a] Deqiang Li,[a] Xin Zhang,[a] Xue Xue,[a] Ran Zhang,[a] Di Su,[a] Tony D. James*[a,c,d], Ping Li*[a][e], Xin Wang*[a] and Bo Tang*[a][b]

[a] College of Chemistry, Chemical Engineering and Materials Science, Key Laboratory of Molecular and Nano Probes, Ministry of Education, Collaborative Innovation Center of Functionalized Probes for Chemical Imaging in Universities of Shandong, Institutes of Biomedical Sciences, Shandong Normal University, Jinan 250014, People's Republic of China.
[b] Laoshan Laboratory, 168 Wenhai Middle Rd, Aoshanwei Jimo, Qingdao 266237, Shandong
[c] Department of Chemistry, University of Bath, Bath BA2 7AY, United Kingdom.
[d] School of Chemistry and Chemical Engineering, Henan Normal University, Xinxiang, 453007, P. R. China.
[e] College of Chemistry and Chemical Engineering, Northwest Normal University, Lanzhou 730070, People's Republic China.

*Tony D. James, Ping Li, Xin Wang, Bo Tang

Email: t.d.james@bath.ac.uk; lip@sdnu.edu.cn; xinwang@sdnu.edu.cn tangb@sdnu.edu.cn

Materials and Methods

Materials and reagents

2,4-dihydroxybenzaldehyde, ethyl 4,4,4-trifluoroacetoacetate, 4-bromomethylphenylboronic acid pinacol ester, triphosgene and DL-norepinephrine hydrochloride were purchased from Shanghai Aladdin Bio-Chem Technology Co., Ltd. Analytical grade solvents were used without further purification. 2-(2-Methoxy-4-nitrophenyl)-3-(4-nitrophenyl)-5-(2,4-disulfophenyl)-2H-tetrazoliumsodiumsalt (CCK-8) was obtained from MedChemExpress. Silicagel plates (HSGF-254 20*20 cm) for TLC were from Yantai Jiangyou silicon development Co., Ltd. PC12 were purchased from Cell Bank of the Chinese Academy of Sciences (Shanghai, China). The C57BL/6J mice (age: 6 weeks; average body weight: 20 ± 2 g) were purchased from Shandong University Laboratory Animal Center. All the animal experiments were carried out in accordance with the relevant laws and guidelines issued by the Ethical Committee of Shandong Normal University (Ethics Approval Number: AEECSDNU2022119).

Reactive oxygen species preparation

Tertbutyl hydroperoxide (TBHP) was diluted from a 70 % aqueous solution. H_2O_2 was diluted from a 30 % aqueous solution. Hypochlorite (NaOCl) was diluted appropriately in 0.1 M NaOH aq. Superoxide ($O_2^{\bullet-}$) was generated from KO_2 in DMSO solution, and the concentration of $O_2^{\bullet-}$ was determined by the concentration of KO_2. Singlet oxygen ($^1O_2$) was prepared using the ClO^-/H_2O_2 system (1:1). Lipid peroxyl radicals were generated via thermolysis of the azo-initiators 2,2'-azobis(2,4-dimethyl)valeronitrile (AMVN) and 2,2'-azobis(2-methylpropionitrile) (ABIN) in acetonitrile solution at 37 °C for 30 min. Hydroxyl radical (•OH) was produced by the reaction of Fe^{2+} with H_2O_2 (1:6). Nitric oxide (NO) was obtained from a stock solution prepared with sodium nitroprusside. Peroxynitrite ($ONOO^-$) was used from a stock solution of 10 mM in 0.3 M NaOH.

Instruments

^1H NMR spectra were obtained at 400 MHz using Bruker NMR spectrometers, and ^{13}C NMR spectra were recorded at 100 MHz. The mass spectra were obtained using the Bruker maXis ultra-high-resolution-TOF MS system. All one-photon (OP) fluorescence measurements were carried out at room temperature on an F-4600 fluorescence spectrometer. The two-photon (TP) excited fluorescence spectra were measured using a Zeiss LSM 880 NLO, and the TP images were acquired with the Zeiss LSM 880 NLO with a 20× water objective. A Ti: sapphire laser was used to excite the specimen at 800 nm with a laser power of 70 mW. The OP images were recorded on a Leica TCS SP8 microscope with a 63× oil-immersion objective (N/A 1.3). The forced swimming tests and tail suspension tests were analysed by DepressionScan (Clever Sys. Inc.). High performance liquid chromatography (HPLC) analysis was carried out by Thermo ScientificTM UltiMateTM 3000.

Cell culture

PC12 cells were purchased from the Cell Bank of the Chinese Academy of Sciences (Shanghai, China), and cultured in DMEM supplemented with 10 % foetal bovine serum, 1 % penicillin and 1 % streptomycin at 37 °C (w/v) in a 5 % CO_2/95 % air MCO-15AC incubator (SANYO, Tokyo, Japan). One day before imaging, the cells were detached and placed in glass-bottomed dishes.

Cytotoxicity assays

The cytotoxicity was measured by 2-(2-Methoxy-4-nitrophenyl)-3-(4-nitrophenyl)-5-(2,4-disulfophenyl)-2H-tetrazoliumsodiumsalt (CCK-8) assay. PC12 cells were seeded in a 96-well plate at a concentration of 1×10^5 cells $well^{-1}$ in 100 μL of RPMI-1640 medium with 10 % fetal bovine serum, 1 % penicillin, and 1 % streptomycin and maintained at 37 °C in a 5 % CO_2 incubator for 12 h. Then, cells were exposed to different concentrations of probe (1×10^{-3}, 1×10^{-4}, 1×10^{-5}, 1×10^{-6}, 1×10^{-7} and 1×10^{-8} M) for 24 h. Then 10 μL CCK-8 was added to each well and continuously

incubated for 1-4 h at 37 °C. During the incubation time, absorbance of each well was measured at 450 nm in a Triturus microplate reader.

High performance liquid chromatography (HPLC)analysis

The parameter of HPLC analytical column used was Acclaim™ 120, C18, 5 μm, 120 Å, 4.6 × 250 mm. Phase A is chromatography grade methyl alcohol, phase B is water, 0.1 M formic acid solution was added into phase A and phase B. The elution ratio of phase A and phase B is 1:1. The flow rate was 1.000mL min^{-1}.

***In vitro* blood–brain barrier (BBB) model**

We established a BBB model in vitro using the transwell cell culture system.[1] Firstly, we inoculated human brain microvascular endothelial cells (hcmec/d3 cells, 2 × 10^5 cells per well) on transwell porous biofilm, and added FBS medium (10%) at the same time. Subsequently, the integrity of the system was evaluated by Millicell-ERS Voltmeter (Millipore). When the resistance was higher than 300 Ω cm^2, the BBB model was successfully constructed for follow-up research. Then, 200 μM Cou-NE-H_2O_2 were added to the upper chamber. After 6 h incubation, the fluorescence intensity of medium in lower chamber in 460 nm was measured.

Mouse models with depression-like behaviours

Adult male C57BL/6J mice (age: 6 weeks; average body weight: 18 ± 2 g) were purchased from the Experimental Animal Center of Shandong University (Jinan, PR China). All animal care and experimental protocols complied with the Animal Management Rules of the Ministry of Health of the People's Republic of China and were approved by the Animal Care Committee of Shandong Normal University. (Ethics Approval Number: AEECSDNU2022119).

The mice were housed in cages under a controlled 12 h/12 h light-dark cycle (lights on: 7:00 a.m.) and given free access to water and food. The mice were allowed to adapt to stable environmental conditions for 1 week and then randomly divided into 2 groups, control and stress. The stress models were established by feeding mice with water added corticosterone. The control group were given water without corticosterone. After 21 days, mice were tested based on the sucrose preference test, forced swimming test, tail suspension test and open field test to prove the stress model was established successfully. For all animal studies, the tester was blind to the group allocation.

Sucrose preference test

Sucrose preference test was conducted using a two-bottle choice procedure before and after medication.[2] Before the sucrose preference test (SPT), mice were habituated to drink a 1 % sucrose solution for 24 h with two bottles. Then, the sucrose solution was replaced with water for an additional 24 h. At the start of the test, mice were given access to the two bottles, one filled with sucrose solution and the other with water. The position of the water and sucrose bottles (left or right) was switched every 30 min for 3 h. Then the mice were left undisturbed, and their overnight fluid consumption was measured at the next morning. The volume of sucrose or water of every bottle was recorded. The sucrose preference was defined as the ratio of the volume of sucrose to the total volume of sucrose and water consumed.

Forced swimming test

Forced swimming test (FST) as a 2 days program were carried out following the literature.[3] In the FST, each mouse was placed in a cylindrical tank (24 cm height × 10 cm diameter) filled to 6 cm with water at a temperature of 24 ± 1 °C. The mice could swim freely. On the first day, the mice exhibited an escape-like behaviour and found an immobile posture that they could maintain their head above water easily for conserving energy. After resting for 24 h, mice could stay immobile rapidly. The mice were subjected to 6 min of swimming, but only the last four minutes were considered in the analysis.

Tail suspension test
In the tail suspension test (TST), each mouse was suspended by the tail using adhesive scotch tape from a hook connected to a strain gauge that detected all the movements of the mouse and transmitted them to a central unit, which calculated the total duration of immobility during a 6 min test. However, only the last four minutes were considered in the analysis.[47]

Open field test
In the open field test (OPT), each mouse was placed in the centre of a test box (40 cm length × 40 cm width × 40 cm height). At the same time, the camera was turned on to record the movement track of the mice in the box, and behavioural analysis software was used to analyze the behavioural indicators such as movement distance, movement speed, and rest time. However, only the first five minutes were considered in the analysis.

Fluorescence imaging experiments
Living PC12 cells were detached and reseeded onto 15 mm glass bottomed dishes 24 h before imaging. Then Cou-NE-H_2O_2 was added and the cells were incubated for 30 min, the cell culture medium was removed, and the cells were washed with 1.0 mL of PBS three times. Living cells fluorescence imaging was performed using a Leica SP8 fluorescence microscope with a blue channel (λ_{ex}=405 nm and λ_{em}=440-480 nm).

For *in vivo* imaging experiments, firstly, we established depression mouse model by administering CORT for 21 days. Mice were separated into control and depressed populations based on the sucrose preference test, forced swimming test, tail suspension test and open field test. Then, 0.34 mg kg^{-1} Cou-NE-H_2O_2 was intraperitoneally injected into both the control mice and the depressed mice, with three mice in each group. After 30 minutes, the mice were euthanized by cervical dislocation, and their brains were extracted to prepare 50 μm-thick tissue sections. These sections were then imaged using a Leica SP8 fluorescence microscope with a blue channel (λ_{ex} = 405 nm and λ_{em} = 440-480 nm).

Transcriptional analysis
Mice in the control group, the stress group and the treatment group were euthanized by cervical dislocation, taking out their brains, quickly cleaning them with sterile, enzyme-free water, and then freezing them with liquid nitrogen for use. Three mice in each group. Then Total RNA was extracted from the mouse brains to detect the integrity and purity of RNA and accurately quantify the total amount of RNA. Then, the cDNA library was constructed and quantified by enriching and fragmenting the mRNA. Finally, the sequencing was performed on the computer, and the experimental results were analyzed using GO functional analysis, KEGG enrichment analysis, GSEA enrichment analysis, and other methods.

Statistical analysis
All data are expressed as the mean ± S.D. The data under each condition were accumulated from at least three independent experiments. For each experiment, unless otherwise noted, n represents the number of individual biological replicates. For each biological replicate and for all in vitro and ex vivo studies, $n \geq 3$. The statistical analyses were performed using Student's t-test.

Data availability
All relevant data that support the findings of this study are available from the corresponding author upon reasonable request.

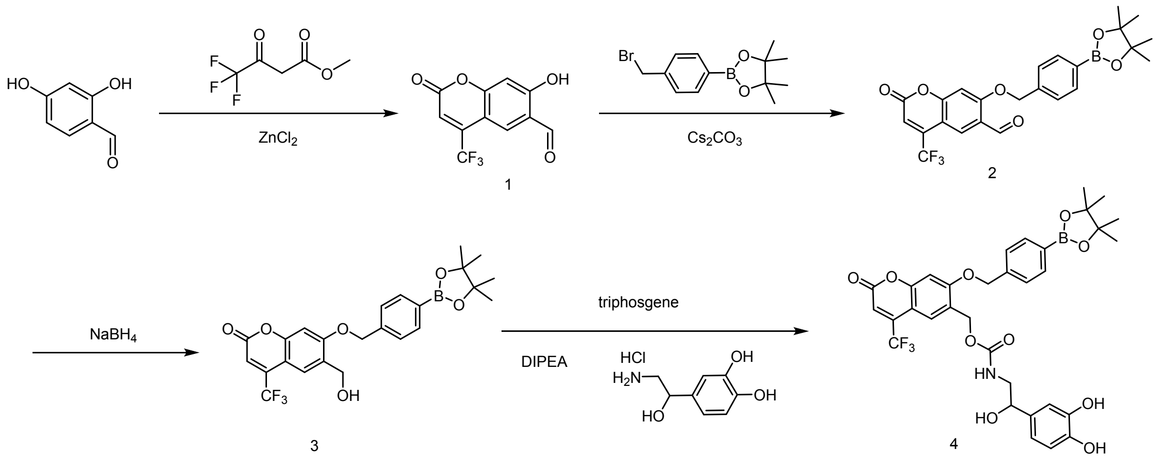

Scheme S1. Synthesis route of Cou-NE-H_2O_2.

Synthesis of Compound 1. Under nitrogen protection, 2,4-dihydroxybenzaldehyde (1.38 g, 10 mmol) and $ZnCl_2$ (0.40 g, 3 mmol) was added to 20 ml ethyl alcohol, followed by the addition of ethyl 4,4,4-trifluoroacetoacetate (1.84 g, 10 mmol).[6] The mixture was refluxed at 80 ℃ for 12 h, then allowed to cool down to room temperature. The resulting product was dried in vacuum to provide the crude product as a pale yellow solid, which was used for the next step directly. HRMS (ESI), m/z calcd for $C_{11}H_5F_3O_4$ $[M-H]^-$ 257.0056, found 257.0011.

Synthesis of Compound 2. Under nitrogen protection, compound 1 (1.29 g, 5 mmol) and 4-bromomethylphenylboronic acid pinacol ester (1.49 g, 5 mmol) were added to 20 mL of acetonitrile, then cesium carbonate (2.61 g, 8 mmol) was added, and the mixture refluxed at 80 °C for 18 hours. The resulting residue was evaporated under reduced pressure, then purified by column chromatography (silica gel, EtOAc/n-hexane, 1/1, v/v) to give compound 2 (1.38 g, 2.9 mmol, 58 % yield) as light yellow oily product. HRMS (ESI), m/z calcd for $C_{24}H_{22}BF_3O_6$ $[M+H]^+$ 475.1538, found 475.1109. ^{1}H NMR (400 MHz, DMSO-d_6) δ 10.01 (s, 1H), 7.70 (d, J = 7.7 Hz, 2H), 7.62 (d, J = 8.7 Hz, 1H), 7.45 (d, J = 7.7 Hz, 2H), 6.64 (dd, J = 8.7, 2.4 Hz, 1H), 6.55 (d, J = 2.4 Hz, 1H), 5.21 (s, 2H), 1.29 (s, 12H). ^{13}C NMR (101 MHz, DMSO-d_6) δ 191.59, 165.31, 163.50, 140.08, 135.09, 134.76, 132.70, 128.81, 128.77, 128.28, 127.89, 127.34, 127.10, 116.86, 108.43, 102.25, 84.14, 69.86, 25.11. ^{11}B NMR (128 MHz, DMSO-d_6) δ 32.73. ^{19}F NMR (376 MHz, DMSO-d_6) δ 71.07.

Synthesis of Compound 3. Under nitrogen protection, compound 2 (0.47 g, 1 mmol) was dissolved in a mixture of anhydrous THF (5 ml) and methanol (3 ml), followed by the slow addition of $NaBH_4$ (1.13 g, 3 mmol), then stirred at room temperature for 3 hours. After the reaction was complete, methanol is slowly added to the reaction system until no bubbles are generated. The resulting product was dried under vacuum to provide the crude product as a pale yellow solid, which was used for the next step directly.

Synthesis of Compound 4. To a solution of compound 3 (0.024 g, 0.05 mmol) in anhydrous THF (15 mL) at 0 °C under N_2 was added triphosgene (0.15 g, 0.5 mmol, 15 wt. % in toluene).[7] The reaction was stirred at room temperature for 3 hours, then N_2 was bubbled through reaction for 20

minutes to obtain the intermediate. The reaction mixture was then added to a solution of DL-norepinephrine hydrochloride (0.10 g, 0.5 mmol) and DIPEA (87.7 μL, 0.5 mmol) in DMF (30 mL) at 0 °C. After 5 hours, the resulting residue was evaporated under reduced pressure, then purified by a column chromatography (silica gel, EtOAc/n-hexane, 1/1, v/v) to give compound 4 (4.02 mg, 0.006 mmol, 12 % yield) as a light yellow soild. HRMS (ESI), m/z calcd for $C_{33}H_{33}BF_3NO_{10}$ $[M-H]^-$ 669.2102, found 669.2564. ^1H NMR (400 MHz, DMSO-d_6) δ 7.67 (q, *J* = 7.9, 7.2 Hz, 2H), 7.58 (d, *J* = 7.6 Hz, 3H), 7.45 (d, *J* = 6.7 Hz, 2H), 7.30 (d, *J* = 7.7 Hz, 1H), 7.23 (d, *J* = 7.8 Hz, 1H), 7.19 (d, *J* = 7.6 Hz, 2H), 5.19 – 5.07 (m, 1H), 4.77 (q, *J* = 11.9 Hz, 1H), 4.03 (q, *J* = 7.0 Hz, 1H), 3.84 (q, *J* = 7.1 Hz, 1H), 2.90 – 2.81 (m, 3H), 2.61 (t, *J* = 7.6 Hz, 1H), 1.28 (s, 12H). ^{13}C NMR (101 MHz, DMSO-d_6) δ 172.51, 167.43, 144.58, 135.00, 134.90, 134.87, 132.43, 131.98, 130.05, 129.13, 128.30, 127.39, 127.06, 84.14, 83.98, 74.36, 71.85, 65.55, 65.48, 61.16, 60.30, 35.26, 34.84, 30.88, 30.47, 29.48, 25.13. ^{11}B NMR (128 MHz, DMSO-d_6) δ 31.03. ^{19}F NMR (376 MHz, DMSO-d_6) δ 76.06.

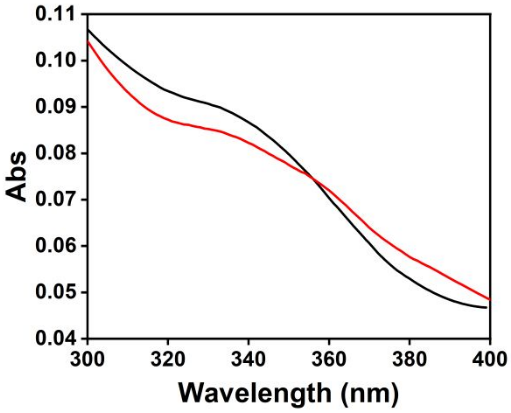

Fig. S1. UV-vis absorptions of Cou-NE-H_2O_2 before and after reaction with H_2O_2. Black line: 20 μM Cou-NE-H_2O_2, red line: 20 μM Cou-NE-H_2O_2 with the addition of 50 μM H_2O_2.

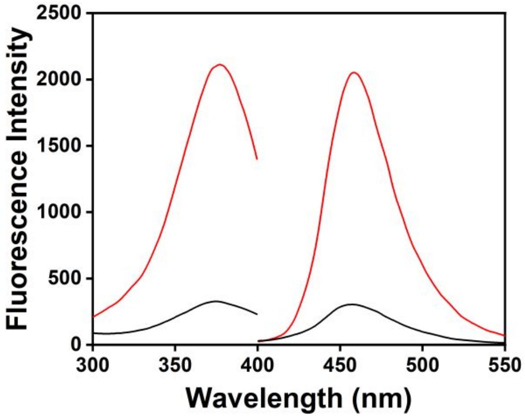

Fig. S2. The excitation and emission fluorescence spectra of Cou-NE-H_2O_2 towards to H_2O_2. Black line: 20 μM Cou-NE-H_2O_2, red line: 20 μM Cou-NE-H_2O_2 with the addition of 50 μM H_2O_2.

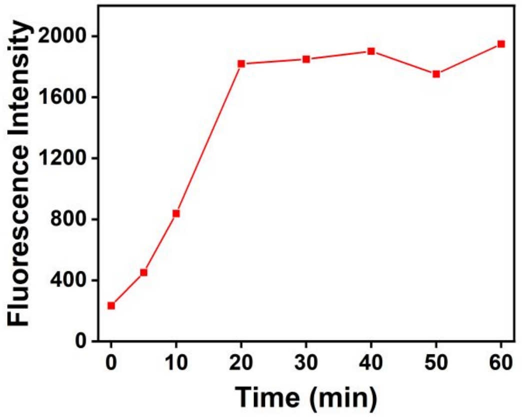

Fig. S3. Kinetic experiments of Cou-NE-H_2O_2 towards to H_2O_2. 20 μM Cou-NE-H_2O_2 reacted with 50 μM H_2O_2 at pH 7.4 in HEPES buffer. $\lambda_{ex}/\lambda_{em}$ = 375/460 nm.

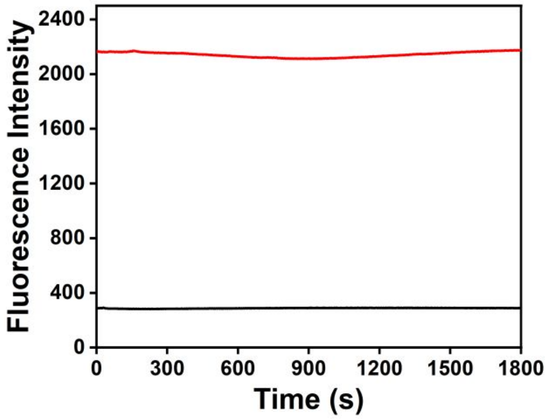

Fig. S4. Photostability of 20 μM Cou-NE-H_2O_2 (black line) alone and after the addition of 50 μM H_2O_2 (red line) at pH 7.4 in HEPES buffer. $\lambda_{ex}/\lambda_{em}$ = 375/460 nm.

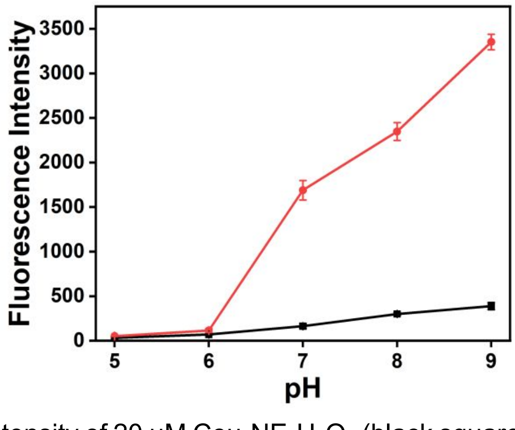

Fig. S5. Fluorescence intensity of 20 μM Cou-NE-H_2O_2 (black squares) alone and after the addition of 50 μM H_2O_2 (red dots) in the presence of a solution with various pH values at λ_{ex} = 375 nm and λ_{em}=460 nm.

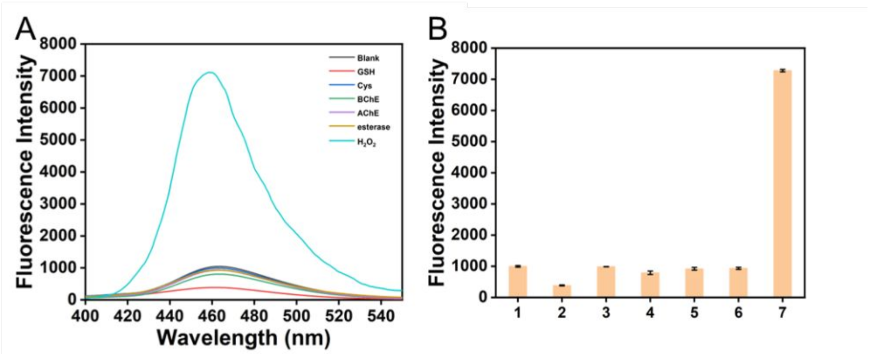

Fig. S6. (A) Fluorescence spectra of 20 μM Cou-NE-H_2O_2 to GSH, Cys, butyrylcholine esterase, acetylcholine esterase and esterase. (B) The output of A. (1. Blank, 2. 1 mM GSH, 3. 100 μM Cys, 4. 10 U/mL butyrylcholine esterase, 5. 100 U /mL acetylcholine esterase, 6. 10 U/mL esterase, 7. 50 μM H_2O_2).

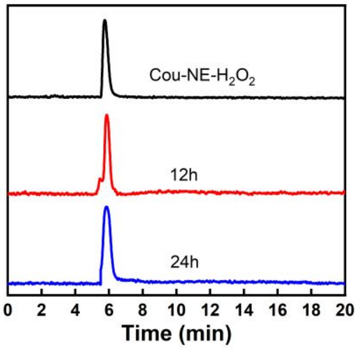

Fig. S7. The HPLC of Cou-NE-H_2O_2 in mouse serum. Black: 20 μM Cou-NE-H_2O_2 was added into 20% mouse serum. Red: 20 μM Cou-NE-H_2O_2 was added into 20% mouse serum for 12h. Blue: 20 μM Cou-NE-H_2O_2 was added into 20% mouse serum for 24h.

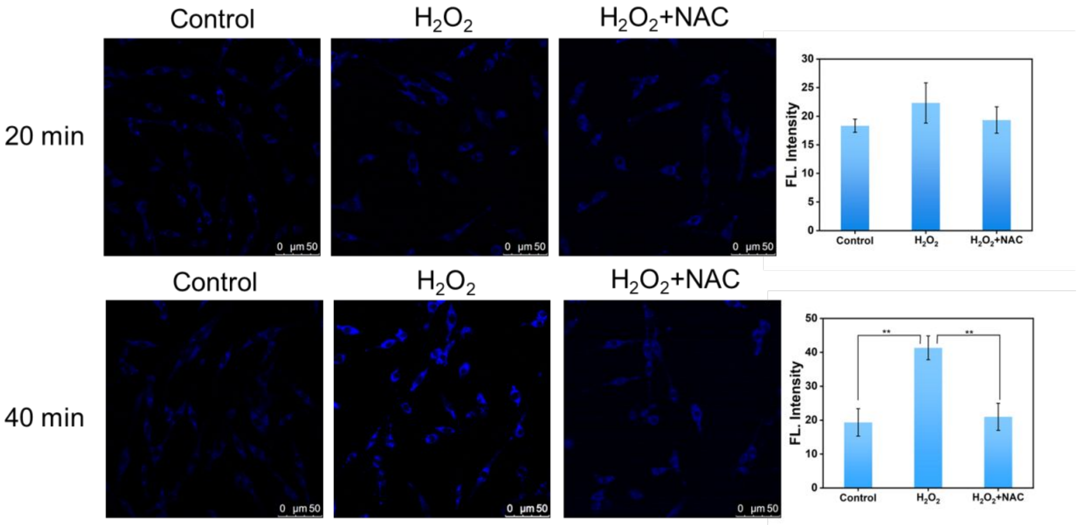

Fig. S8. Confocal fluorescence imaging of PC12 cells at different incubation time. Control: PC12 cells were incubated with 20 μM Cou-NE-H_2O_2 for 20 min or 40 min respectively. H_2O_2: PC12 cells were pretreated with 200 μM H_2O_2 for 30 min and then incubated with 20 μM Cou-NE-H_2O_2 for 20 min or 40 min respectively. H_2O_2+NAC: PC12 cells were pretreated with 200 μM H_2O_2 for 30 min and loaded with 200 μM NAC for 30 min, then cells were incubated with 20 μM Cou-NE-H_2O_2 for

20 min or 40 min respectively. The right column shows the output of fluorescence intensity. The images were acquired at an excitation wavelength of 405 nm and at emission wavelengths of 440-480 nm. Scale bar = 100 μm. The data are expressed as mean ± S.D, n=3. ***P < 0.001 compared to the control group.

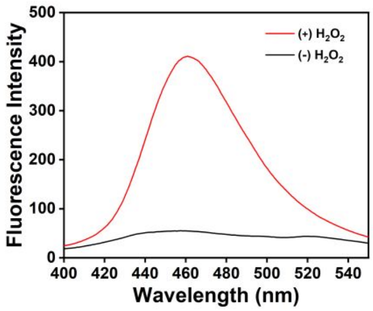

Fig. S9. Fluorescence spectra of Cou-NE-H_2O_2 in medium. Red: Fluorescence spectrum of the medium below transwell chamber after adding H_2O_2. Black: Fluorescence spectrum of the medium below transwell chamber without H_2O_2.

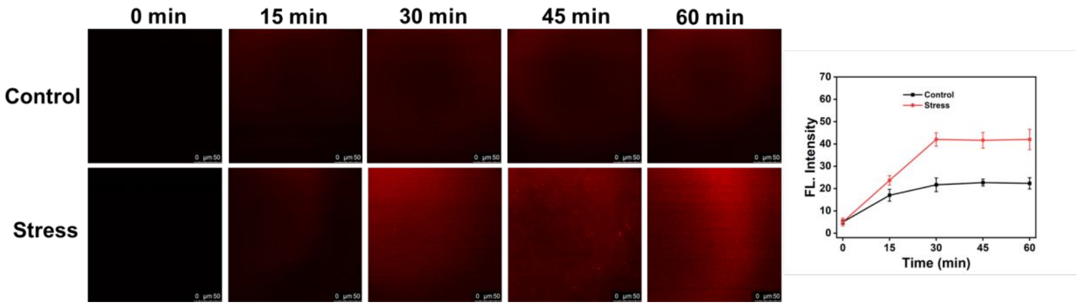

Fig. S10. Time-dependent fluorescence imaging of mouse brains. Normal mice and depressed mice were euthanized at 0, 15, 30, 45 and 60 minutes after the intraperitoneal injection of Cou-NE-H_2O_2. The brains were removed for frozen section, and then fluorescent imaging was performed on the brain tissue sections using a Leica SP8 fluorescence microscope with a blue channel (λ_{ex} = 405 nm and λ_{em} = 450-470 nm). Three mice in each group, the thickness of brain tissue section is 50 μm. Scale bar = 50 μm. The right column is the output of the fluorescence images.

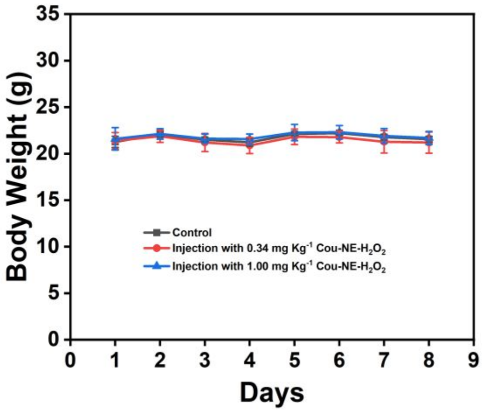

Fig. S11. The biocompatibility of Cou-NE-H_2O_2. Black line: mice injected with 0.9 % NaCl aqueous solution. Red line: mice injected with 0.34 mg Kg^{-1} Cou-NE-H_2O_2. Blue line: mice injected with higher concentration 1.00 mg Kg^{-1} Cou-NE-H_2O_2. The experimental data showed the body weights of treated groups were identical to control group over one week. 5 mice in each group.

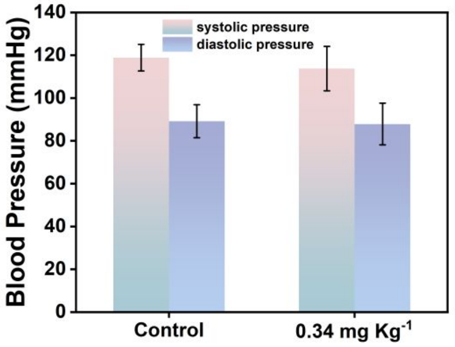

Fig. S12. The blood pressure of mice injected with Cou-NE-H_2O_2 for 2 weeks. Control: mice injected with 0.9 % NaCl aqueous solution. 0.34 mg Kg^{-1}: mice injected with 0.34 mg Kg^{-1} Cou-NE-H_2O_2.

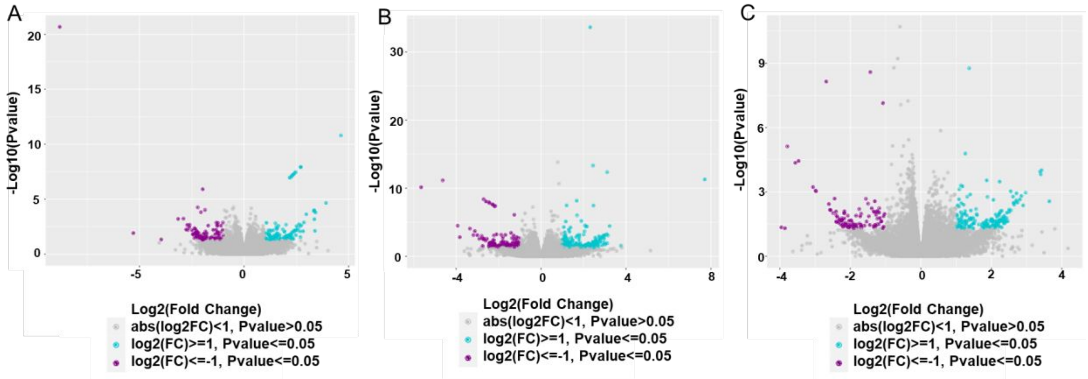

Fig. S13. Genetic volcano maps. (A) Stress vs Control. (B) Treatment vs Stress. (C) Treatment vs Control. Purple represents significantly down-regulated genes, blue represents significantly up-regulated genes, and gray represents non-significantly differenced genes.

References

[1] T. R. Powell, C. Fernandes, L. C. Schalkwyk, *Curr. Protoc. Mouse Biol.* **2012**, *2*, 119-127.
[2] B. Petit-Demouliere, F. Chenu, M. Bourin, *Psychopharmacology (Berl)* **2005**, *177*, 245-255.
[3] H. C. Yan, X. Cao, M. Das, X. H. Zhu, T. M. Gao, *Neurosci. Bull.* **2010**, *26*, 327-337.

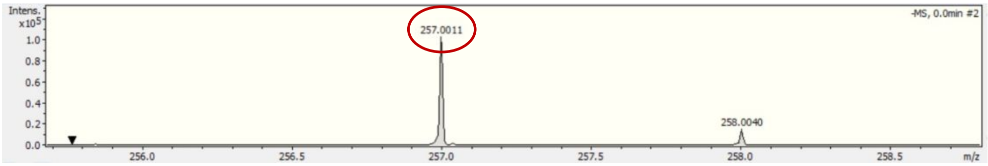

Fig. S14. HRMS of Compound 1.

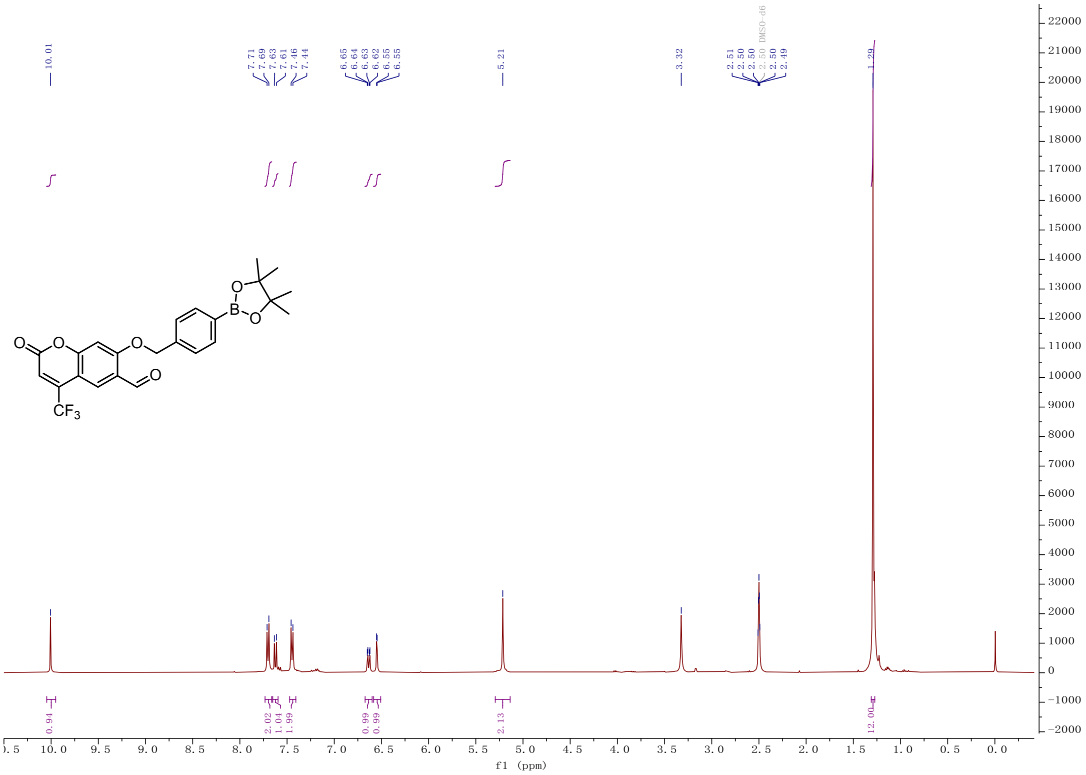

Fig. S15. ^{1}HNMR of Compound 2.

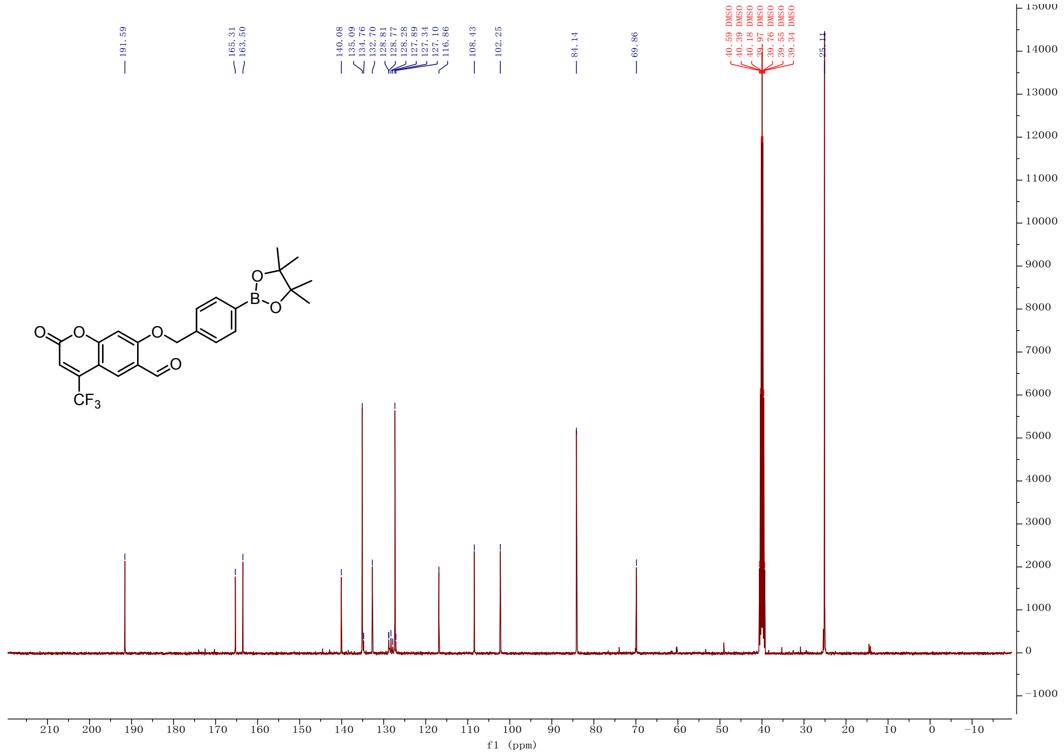

Fig. S16. ^{13}CNMR of Compound 2.

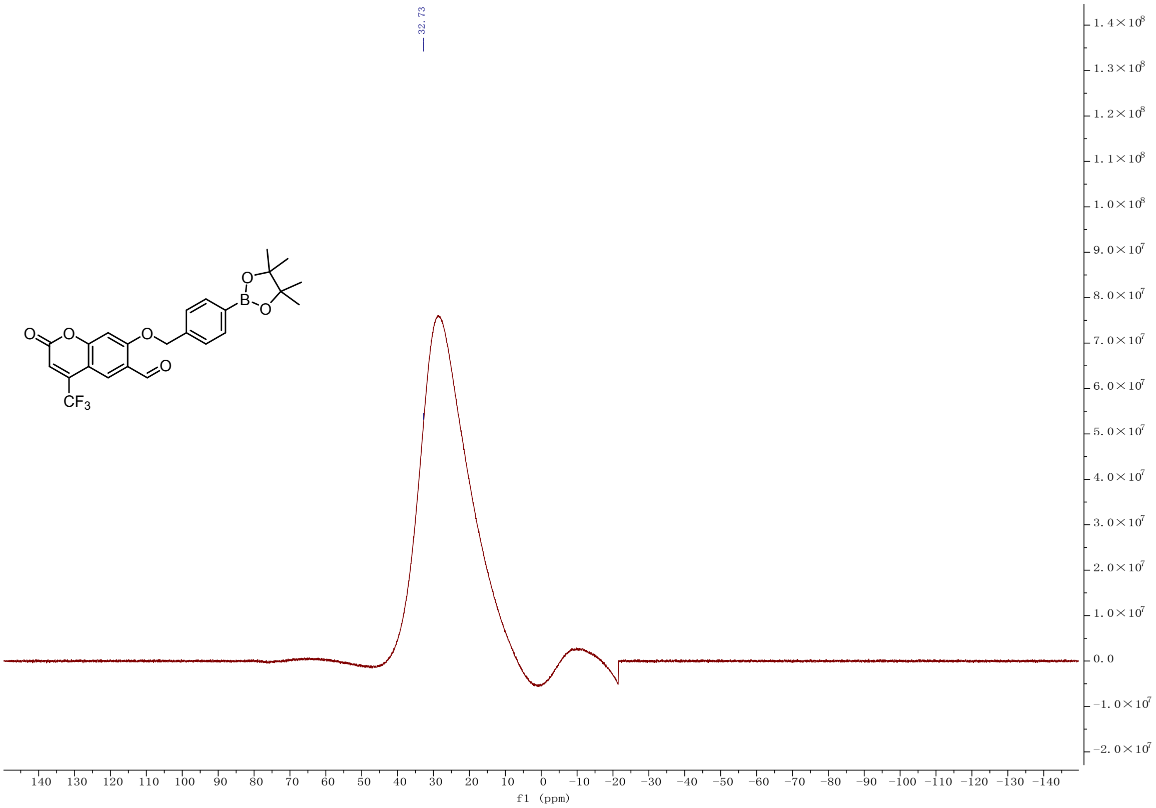

Fig. S17. ^{11}BNMR of Compound 2.

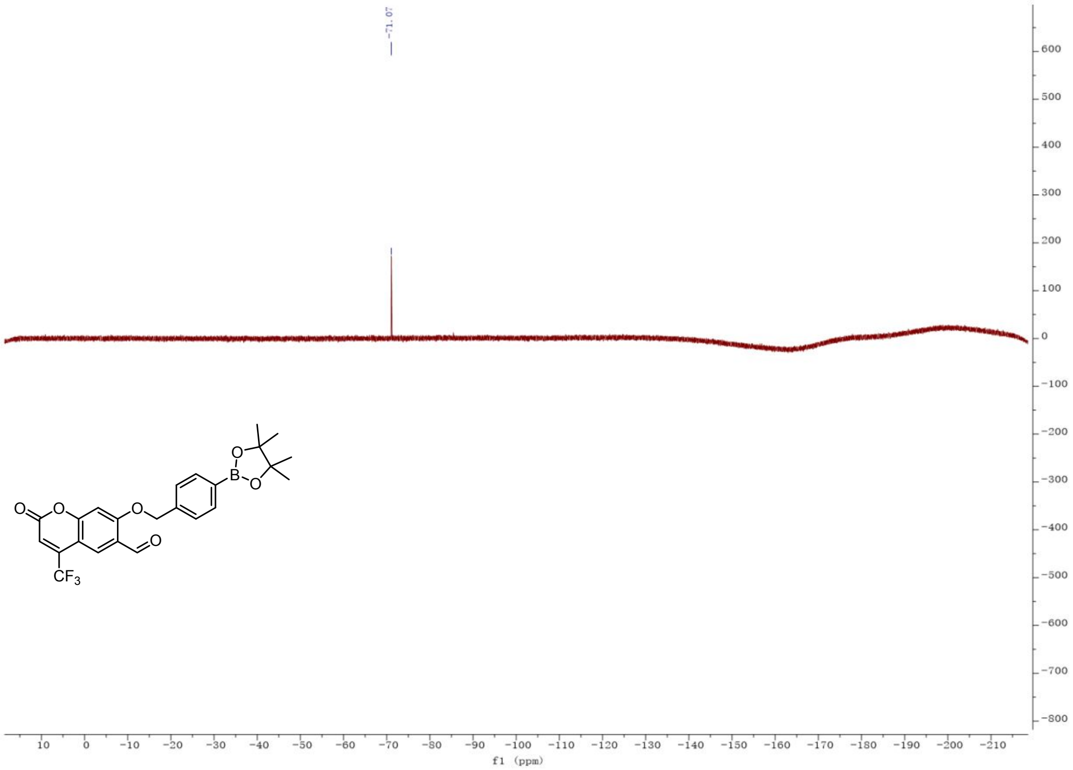

Fig. S18. ^{19}FNMR of Compound 2.

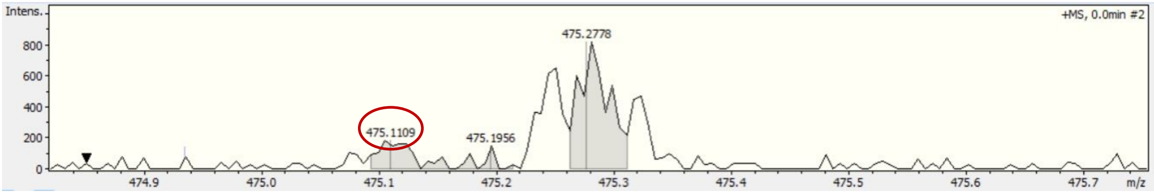

Fig. S19. HRMS of Compound 2.

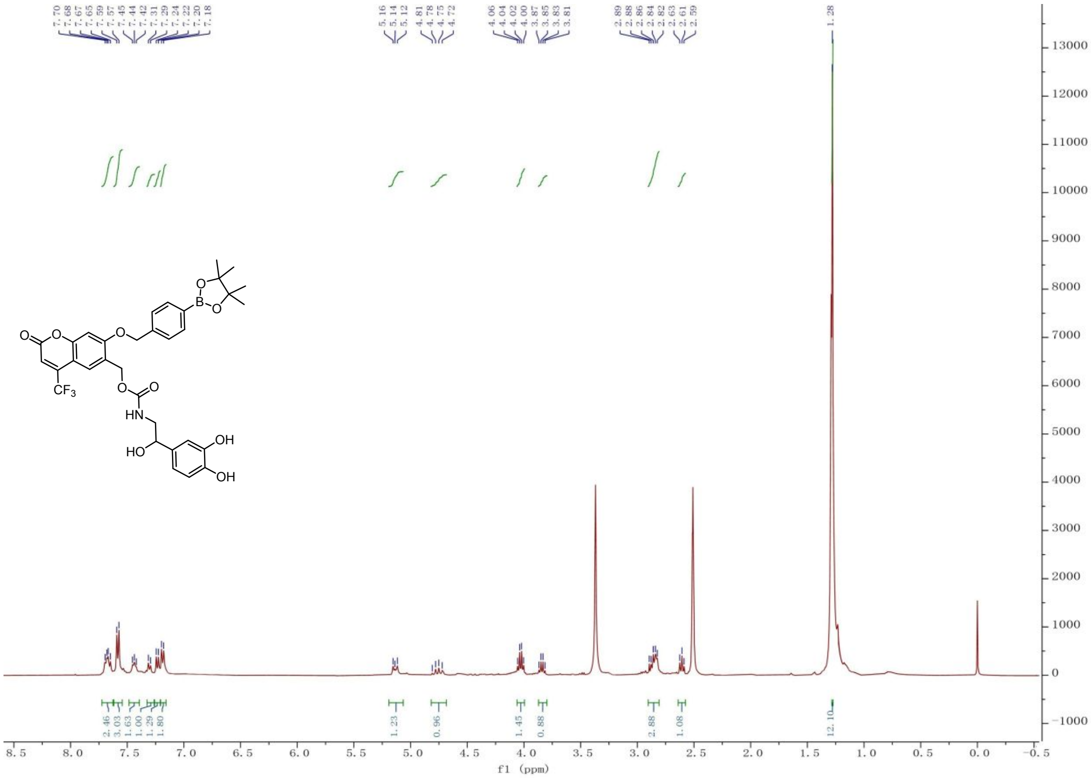

Fig. S20. ^{1}HNMR of Compound 4.

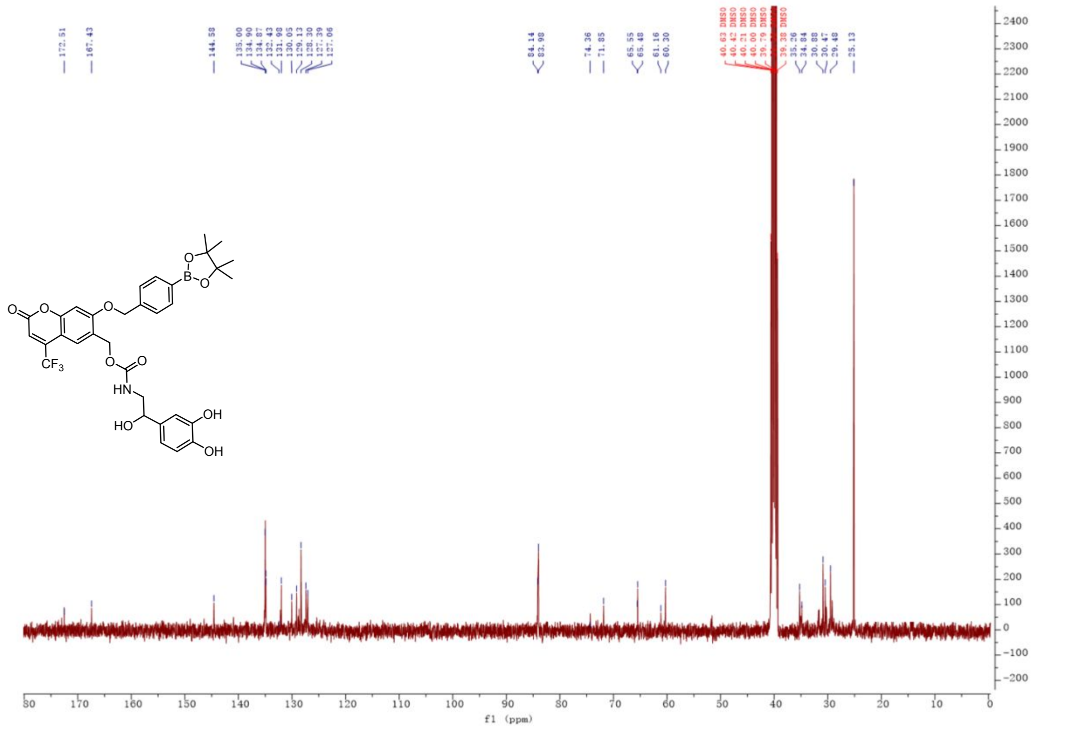

Fig. S21. ^{13}CNMR of Compound 4.

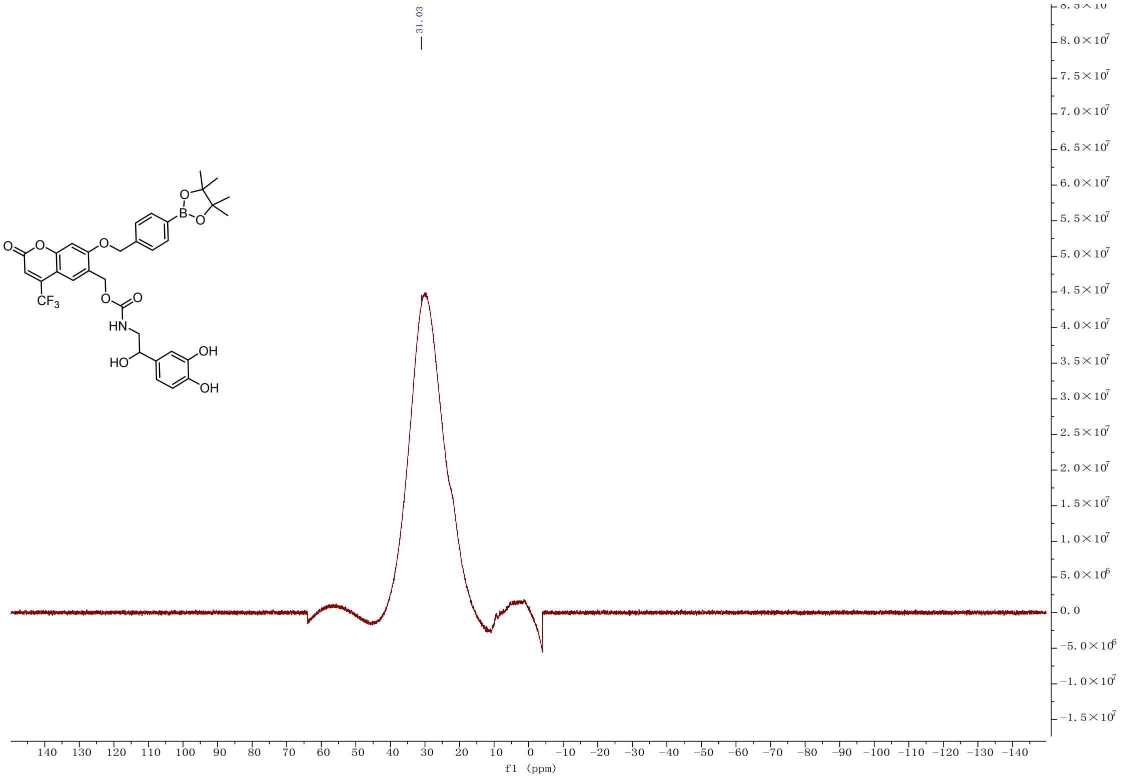

Fig. S22. ^{11}BNMR of Compound 4.

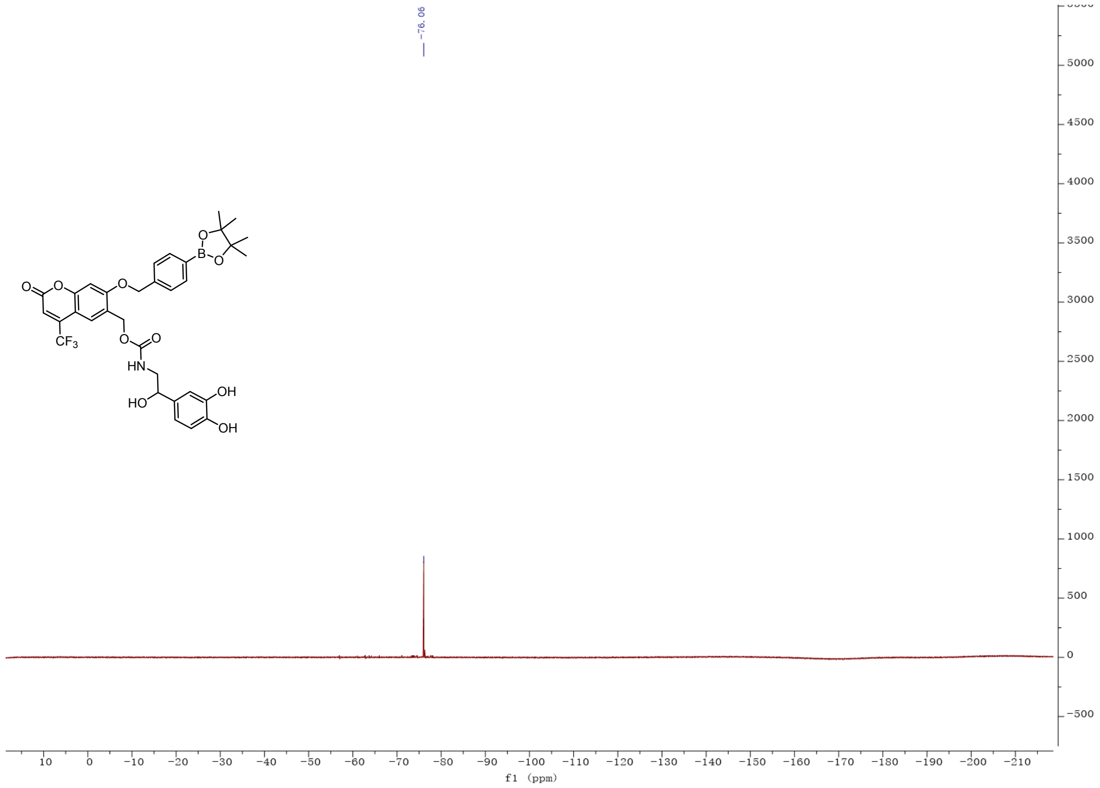

Fig. S23. ^{19}FNMR of Compound 4.

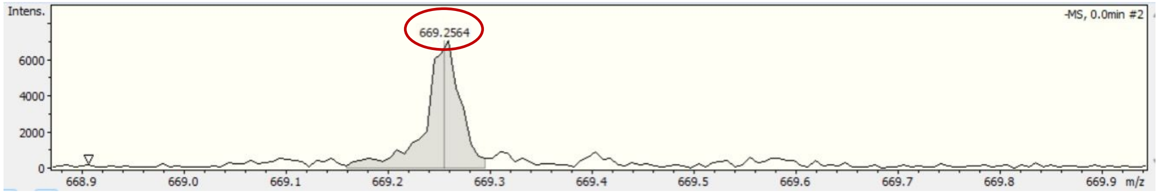

Fig. S24. HRMS of Compound 4.
